# Knock-in of Mutated *hTAU* Causes Insulin Resistance, Inflammation and Proteostasis Disturbance in a Mouse Model of Frontotemporal Dementia

**DOI:** 10.1007/s12035-019-01722-6

**Published:** 2019-08-08

**Authors:** Claire Hull, Ruta Dekeryte, David J. Koss, Barry Crouch, Heather Buchanan, Mirela Delibegovic, Bettina Platt

**Affiliations:** Institute of Medical Sciences School of Medicine, Medical Sciences & Nutrition Foresterhill, University of Aberdeen, Aberdeen, Scotland AB25 2ZD UK

**Keywords:** Transgenic, Knock-in, Diabetes, Insulin, Glucose, ER stress, UPR, Proteinopathy

## Abstract

Diabetes and obesity have been implicated as risk factors for dementia. However, metabolic mechanisms and associated signalling pathways have not been investigated in detail in frontotemporal dementia. We therefore here characterised physiological, behavioural and molecular phenotypes of 3- and 8-month-old male tau knock-in (PLB2_TAU_) vs wild-type (PLB_WT_) mice. Homecage analysis suggested intact habituation but a dramatic reduction in exploratory activity in PLB2_TAU_ mice. Deficits in motor strength were also observed. At 3 months, PLB2_TAU_ mice displayed normal glucose handling but developed hyperglycaemia at 8 months, suggesting a progressive diabetic phenotype. Brain, liver and muscle tissue analyses confirmed tissue-specific deregulation of metabolic and homeostatic pathways. In brain, increased levels of phosphorylated tau and inflammation were detected alongside reduced ER regulatory markers, overall suggesting a downregulation in essential cellular defence pathways. We suggest that subtle neuronal expression of mutated human tau is sufficient to disturb systems metabolism and protein handling. Whether respective dysfunctions in tauopathy patients are also a consequence of tau pathology remains to be confirmed, but could offer new avenues for therapeutic interventions.

**Electronic supplementary material:**

The online version of this article (10.1007/s12035-019-01722-6) contains supplementary material, which is available to authorized users.

## Introduction

Alzheimer’s disease (AD) is the most common form of dementia, characterised by a progressive loss of memory and decline in cognitive function. Histopathologically, end-stage AD is defined by two extracellular aggregates, β-amyloid and neurofibrillary tangles (NFTs). Though both pathologies must be present for a diagnosis of AD, it is important to note that NFTs correlate better with cognitive decline and disease duration and tau-only pathology is observed in isolation, for example in frontotemporal dementia (FTD; [1]).

The microtubule-associated protein tau is essential for cytoskeletal stability and assembly, its biological activity is regulated by post-translational modifications, e.g. abnormally hyperphosphorylated tau is detrimental to neuronal function and ultimately leads to paired helical filaments (PHFs), the main constituents of NFTs. Hyperphosphorylation of tau may also promote conformational changes, which can lead to seeding and spreading of pathological protein species throughout the brain [2].

Considering the heterogeneity between and within the different types of dementia, it appears likely that tau pathology may initially be caused by a complex interplay between several multifactorial components, on the background of an ageing brain. To date, there is increasing evidence supporting a link between neurodegenerative disorders and metabolic dysfunction, including glucose intolerance and insulin resistance [3]. A number of studies have examined the association between type 2 diabetes (T2D) and FTD, with some suggesting that impaired insulin secretion, glucose intolerance and hyperglycaemia are risk factors [3]. Peripheral insulin resistance is a central feature of diabetes, but increasing evidence suggests that *neuronal* insulin signalling also regulates cognitive function [4, 5]. Conversely, impaired tau function may contribute to metabolic changes seen in dementia patients. Accordingly, a recent study demonstrated that deletion of tau can lead to impaired hippocampal responses to insulin via alterations in IRS1 (insulin receptor substrate 1) and PTEN (phosphatase and tensin homologue) signalling [5]. Another report found that knockout of the insulin receptor (IR) resulted in a substantial increase in hyperphosphorylated tau at sites associated with neurodegenerative diseases [6]. For these observations, cellular dyshomeostasis such as ER stress may be the common denominator, since ER disruptions occur in both metabolic and neurodegenerative disease, and polymorphisms in ER key mediators are key risk factors for proteinopathies such as AD and FTD [7, 8]. Specifically, the ER plays a central role in proteostasis [9] and responses to physiological challenges. Hence, a range of pathological and age-related stimuli can disrupt ER homeostasis resulting in accumulation of unfolded or misfolded proteins. Cerebral proteinopathies and also T2D are characterised by disturbances in respective restorative ER function, with the ‘unfolded protein response’ (UPR) alleviating stress during an acute high turnover of proteins. However, chronic activation of the UPR can lead to cell dysfunction and apoptosis [10], and hence, restoration of normal ER function may offer a potential therapeutic approach for various diseases including FTD and T2D [11–13]. Importantly, a recent study in a mouse model of frontotemporal dementia (Tg4510) showed that treatment with the PERK inhibitor GSK2606414 restored protein synthesis rates, protected against neuronal loss and reduced levels of ER stress markers phospho (p)-PERK, p-eIF2α and ATF4 [12]. Similarly, another study in a T2D model utilised the ER chaperone tauroursodeoxycholic acid (TUDCA), which led to normalisation of hyperglycaemia, enhanced insulin action and restoration of insulin sensitivity [14]. Overall, the UPR is implicated in both FTD and T2D relevant disease processes [15], but its exact function is not fully elucidated yet [16].

Here, we sought to investigate the role of central vs peripheral insulin signalling and ER stress pathways in a murine knock-in model of frontotemporal dementia (FTD), termed PLB2_TAU_. These mice express a single copy of mutated hTau (2N4R Tau_P301L + R406W_), which yields brain phospho-tau pathology and disturbances in activity, cognition and sleep, relevant for an FTD-like phenotype [17]_._ We provide evidence that neuronal metabolic, inflammatory and ER stress pathways are affected by abnormal hTau expression, ultimately leading to a systemic diabetic phenotype and tissue-specific disturbances in insulin signalling, indicative of a crucial role for tau in metabolic and homeostatic regulation.

## Materials and Methods

### Animals

Three-month-old male PLB_WT_ (‘WT’, *n* = 7) and PLB2_TAU_ (*n* = 14) mice and eight-month-old male PLB_WT_ (*n* = 17) and PLB2_TAU_ (*n* = 10) mice were generated as previously described [17]. Of note, the PLB_WT_ mice, which serve as controls for all PLB lines, were originally created as littermates from the parental PLB1_Double_ mice. All PLB lines are maintained at Charles River, UK, on a C57BL6 background, and all lines are regularly crossed with unrelated C57BL6/J (elite breeders from Charles River). All mice were housed and tested in accordance with UK Home Office regulations, i.e. the EU directive 63/2010E and the Animal (Scientific Procedures) Act 1986. Mice were bred (Charles River) and delivered to our facility several weeks before testing. Animals were housed in a climate-controlled holding room (20–21 °C, 60–65%, relative humidity) with ad libitum access to water and food with a 12-h day/night cycle (lights on at 7 am).

### In Vivo Characterisation

#### Glucose, Insulin, Pyruvate Tolerance Tests and EchoMRI

Tail blood glucose was determined using an AlphaTRAK glucometer (Berkshire, UK). Glucose (GTT) and insulin tolerance (ITT) tests were performed in 5-h-fasted mice, and pyruvate tolerance tests (PTT) were performed in 15-h-fasted mice. Body weights and fasting blood glucose (time 0) were recorded, followed by i.p. injection of glucose, insulin or pyruvate: GTT, 2 mg/g body weight dose of glucose (20% w/v glucose); ITT, 0.75 IU/g body weight dose of human insulin (Humulin R; Eli Lilly); PTT, 2 mg/g body weight dose of pyruvate (20% w/v pyruvate). Blood glucose levels were determined at 15, 30, 60 and 90 min post-injection. Body composition (adiposity and lean body mass) was assessed using EchoMRI as described in previous studies (EchoMRI, Houston, TX, USA; [18]).

#### PhenoTyper Home Cage Activity

Locomotor and circadian activity were assessed using the PhenoTyper (Noldus, The Netherlands; [19]) home cage observation system. Activity (distance moved) was recorded for 7 days and data extracted in 1-h or 10-min bins. The first 3 h of recording served as habituation to a new environment. The mean hourly activity was calculated over 24 h. The animals were given free access to weighted amount of food and water during PhenoTyper home cage analysis and the amount of food consumed was recorded.

#### RotaRod

Automated four-lane accelerating RotaRods (UgoBasile NG Rotarod 1.3.2R) were used to examine motor coordination and motor learning. Testing occurred on 2 consecutive days with 4 trials per day (5-min intervals) using an acceleration from 1 to 45 rpm over 5 min.

### Ex Vivo Molecular Characterisation

#### Blood Plasma Collection

Mice were fasted for 5-h and 20 μl of blood was collected from the tail of each mouse into a BD Microtainer SST Tube (BD Biosciences, CA, USA). Tubes were left to coagulate at room temperature for 30-min and centrifuged at 7500 rpm for 15-min at 4 °C. Serum was aliquoted and stored long-term at − 80 °C.

#### Insulin ELISA

An Insulin ELISA (Merck Millipore, Cat No: EZRMI-13K) was used to quantify insulin levels in PLB2_TAU_ mice using blood serum samples from 5-h-fasted mice. This assay was carried out following manufacturers’ instructions and absorbance measured at 450 nm and 590 nm using BMG labtech FLUOstar Omega plate reader (BMG Labtech, Germany).

#### Brain, Muscle and Liver Tissues

Eight-month-old mice were fasted for 5-h and an i.p. injection of insulin (10 mU/g body weight) was administered. Mice were sacrificed by neck dislocation; brains, muscle and liver removed and snap-frozen in liquid nitrogen and stored at − 80 °C for future use. Brain tissue was homogenised in NP40 lysis buffer (1 M HEPES, 5 M sodium chloride (NaCl), 0.1 M ethylenediaminetetraacetic acid (EDTA) (1%), NP-40 (Sigma, Dorset, UK): pH = 7.6). For tau Western blot detection, heat-stable fractions were isolated by further heating the brain supernatant at 90 °C for 10 min before being spun (14,000 rpm, 4 °C, and 10 min) and the resulting supernatant collected. Muscle or liver tissue was homogenised in RIPA lysis buffer (10 mM Tris-HCl, 150 mM NaCl, 0.1% SDS, 1% Triton, 1% sodium deoxycholate, 5 mM EDTA, 1 mM NaF, 1 mM NaOV: pH = 7.4). Both buffers were supplemented (1 tablet/10 ml) with complete protease inhibitors (Roche) and PhosStop tablets (Roche). Homogenates were centrifuged (14,000*g*, 4 °C, 20 min) and the supernatant collected and stored at − 80 °C.

#### Protein Analyses

Sample protein concentration was adjusted using a BCA protein assay (Sigma, Dorset, UK) to a final concentration of 3 μg/μl. Samples were prepared with 15 mM dithiothreitol (DTT; Sigma), lithium dodecyl sulphate (LDS, Thermo Fisher Scientific, Paisley, UK), and NP40 lysis buffer and were boiled for 10 min at 70 °C and separated on a pre-cast NuPage 4–12% sodium Bis-Tris electrophoresis gels. Electrophoresis was conducted for 45 min at 200 V in MOPS buffer. Proteins were transferred onto a nitrocellulose (0.45-μm pore size, Invitrogen, UK) membrane using wet transfer (NuPage transfer buffer in dH_2_O with 10% methanol) at 25 V for 1 h. Tris-buffered saline with Tween (TBST) (0.05% Tween, 50 mM Trizma base, 150 mM NaCl) was used for washing (3 × 15-min washes). After transfer, membranes were blocked (5% milk powder in TBST) for 1 h at RT. Subsequently, membranes were washed in TBST (3 × 10 min) and incubated overnight at 4 °C in primary antibodies. Primary antibodies (Suppl. Table 1) were prepared using 5% BSA, 0.05% sodium azide and TBST. The following day, membranes were washed and incubated in appropriate secondary antibodies (Suppl. Table 1). Western blots were visualised using freshly prepared enhanced chemiluminescent substrate (ECL; 0.015% hydrogen peroxide (H_2_O_2_), 30 μM coumeric acid in 1.25 mM luminol). Images were captured using a Vilber-Fusion chemiluminescence-imaging camera and Fusion Software (PEQLAB).

#### Quantification

Coomassie Blue was used as a protein loading control for Western blots as described previously [20]. All loading control images are provided as a supplementary document (Suppl.1). Densitometric analysis of 16-bit Western blots images was performed using ImageJ (NIH) software. Data for phospho markers were first normalised to total protein prior to expression relative to WT controls; all other markers were normalised to total protein and expressed relative to controls.

### Puromycin Assay

Male PLB_WT_ (*n* = 12) and PLB2_TAU_ (*n* = 9) mice (8 months old) were anaesthetised and unilateral intracerebroventricular injections perfromed of either puromycin (25 mg/2.0 ml; Sigma, Dorset, UK), vehicle (10% DMSO in artificial cerebrospinal fluid, aCSF), negative control (puromycin and thapsigargin (0.5 μg/μg; Tocris, Abingdon, UK)); coordinates: − 2 mm anterioposterior, 2 mm mediolateral, and 1.5 mm dorsoventral), as described previously [21]. Mice were sacrificed by cervical dislocation 2 h after the injection. The cerebellum was removed, and half brains dissected for total protein extraction. Incorporation of puromycin was detected via Western blots as described above, using overnight incubation with an anti-puromycin antibody (1:5000; Millipore), prior to secondary antibody labelling, imaging and quantification.

### Quantitative Polymerase Chain Reaction (qPCR)

Total RNA was isolated from cortical and liver mouse tissue using TRIReagent (Ambion, Warrington, UK) according to the manufacturer’s protocol. cDNA was synthesised from 1 μg of total RNA using the bioline cDNA synthesis kit (Bioline, London, UK). Target genes were amplified by quantitative polymerase chain reaction (PCR) using GoTaq qPCR Master Mix (Promega, Southampton, UK) in a Roche LightCycler® 480 System (Roche Diagnostics, Burgess Hill, UK). Relative gene expression was calculated using the comparative Ct method (2−δδCt). Primer sequences used for qPCR are listed in Suppl. Table 2. The geometric mean of three of the most stable reference genes (Y-Whaz, NoNo, 18S, GAPDH or BetaActin) were used to normalise data.

### Primary Hippocampal Cell Culture

Hippocampal cultures from 1- to 3-day-old C57BL/6 mice were prepared as described previously [22]. Briefly, the hippocampus was dissected and dissociated in HEPES-buffered saline (HBS; 130 mM NaCl, 5.4 mM KCl, 1.8 mM CaCL_2_, 1 mM MgCl_2_, 10 mM HEPES, 25 mM d-glucose, pH 7.4) containing 1 mg/ml protease type XIV. Tissue was triturated in HBS, centrifuged and re-suspended in medium. Neurobasal medium was supplemented with 5% foetal bovine serum, 2% B27, 1% l-glutamine and 0.025% glutamate at 37 °C in a humidified atmosphere with 5% CO_2_ and maintained for 5 days. Subsequently, cells were treated with thapsigargin (0.5 μM) for 24 h followed by RNA extraction used as positive control for ER stress.

### Statistical Analysis

Statistical analysis was performed using Prism (V6, GraphPad). Comparisons between two groups applied Student’s two-tailed *t* tests, multiple groups were compared using parametric or non-parametric analysis of variance (one-way or two-way ANOVA) followed by Bonferroni post-tests as appropriate for selected data sets of interest. Nonlinear regression analysis with one-phase decay was used for habituation data. For all data, *p* < 0.05 was considered reliable.

## Results

### Body Weights and Metabolic Phenotypes at 3 Months of Age

**Fig. 1:**
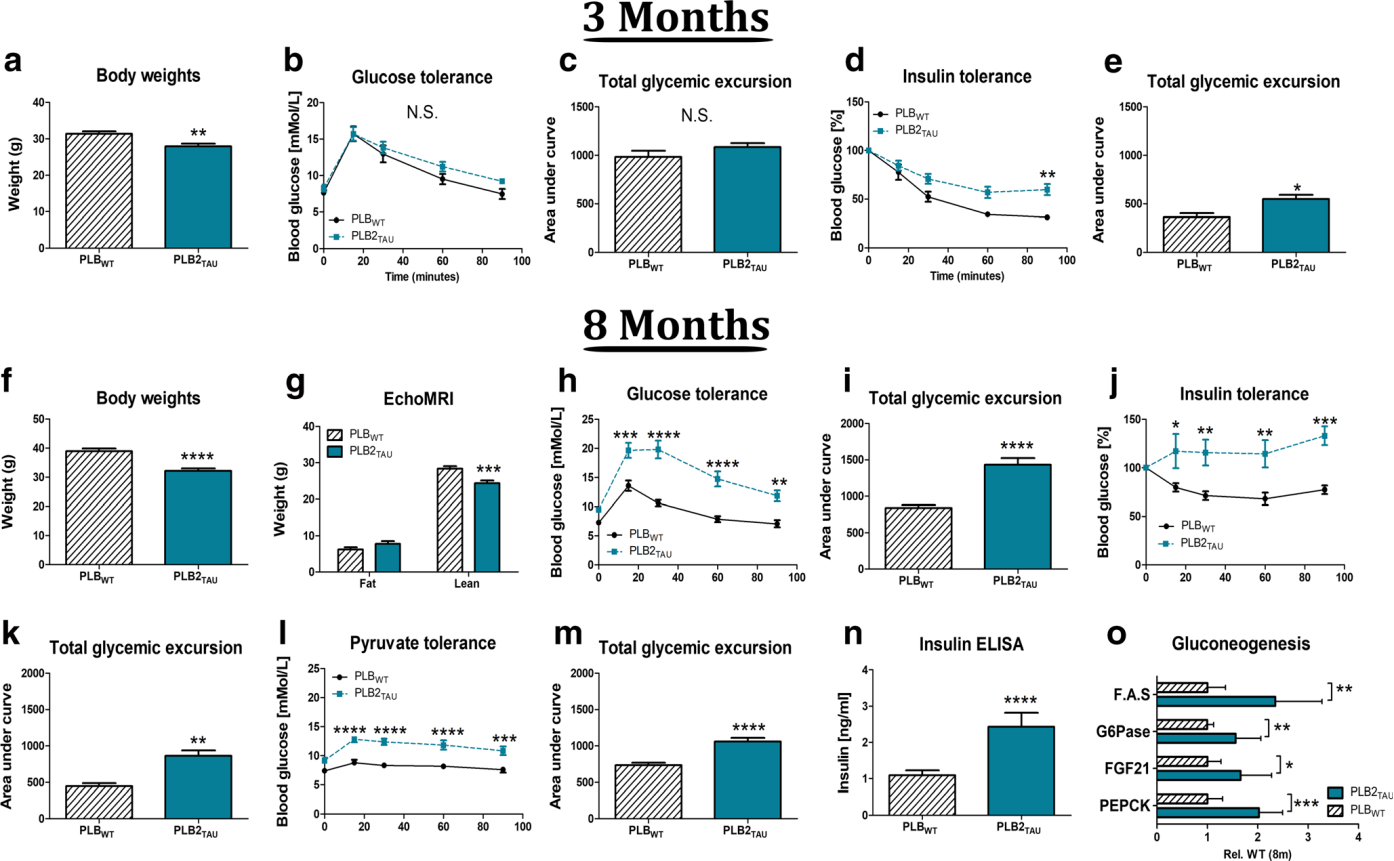
Metabolic phenotype of 3- and 8-month-old male PLB_WT_ (*n* = 17) and PLB2_TAU_ (*n* = 10) mice. Three-month-old (PLB_WT_ (*n* = 7) and PLB2_TAU_ (*n* = 14)): (**a**) Body weights. (**b**, **c**) Glucose tolerance test (GTT) and quantification of area under the curve (AUC) for total glycaemic excursions. **d**, **e**) Insulin tolerance tests (ITTs) and quantification of AUC. 8-month-old: (**f**) Body weights. (**g**) EchoMRI. **h**, **i** GTT and quantification of AUC for total glycaemic excursions. **j**, **k** ITTs and quantification of AUC. **l**, **m** Pyruvate tolerance tests (PTTs) and quantification of AUC. **n** Quantification of insulin levels in serum. **o** Quantification of qPCR gluconeogenesis markers in liver tissue. N.S., not significant. ****p < 0.0001, ***p < 0.001, **p < 0.01, *p < 0.05

Body weights and glucose and insulin intolerance were examined in 3-month-old male PLB2_TAU_ mice and their age-matched controls. PLB2_TAU_ mice weighed less cf. WT (~ − 10%; *p* = 0.009; Fig. 1(a)). A glucose tolerance test revealed normal glucose handling for all time points, with no significant difference in total glycaemic excursion (Fig. 1(b, c)). However, PLB2_TAU_ mice displayed early signs of insulin resistance, i.e. higher blood glucose levels were detected 90 min post bolus injection of insulin (Fig. 1(d)), alongside an overall increase in total glucose excursion (*p* = 0.0178; Fig. 1(e)) compared with WT mice.

### Body Composition and Metabolic Phenotype at 8 Months of Age

Body adiposity, body weights and glucose tolerance were next examined in 8-month-old male PLB2_TAU_ mice. Transgenic PLB2_TAU_ mice continued to weigh less (− 17%; *p* = 0.0001) than PLB_WT_ controls and EchoMRI results indicated this to be due to a lower lean body mass (− 14%; *p* < 0.001) (Fig. 1(f, g)). Furthermore, 5-h-fasted PLB2_TAU_ mice presented with elevated blood glucose levels and severe glucose intolerance over all time points during glucose tolerance tests (GTTs, *p* < 0.001) (Fig. 1(h, i)). Additionally, these mice were also dramatically insulin resistant following a bolus injection of insulin (*p* < 0.05) (Fig. 1(j, k)). Pyruvate tolerance tests (PTTs) were performed in 15-h-fasted mice, since pyruvate bolus injection elicits a glycaemic excursion that reflects hepatic gluconeogenesis (Fig. 1(l, m)). PLB2_TAU_ mice had significantly higher blood glucose levels following the pyruvate challenge during the full 90-min time course (*p* < 0.001), with a significant increase in overall blood glucose levels (AUC; *p* < 0.0001). Basal insulin levels were also determined to be increased in PLB2_TAU_ mice compared with age-matched controls (*p* = 0.0007; Fig. 1(n)). Following this, markers for gluconeogenesis were probed in PLB2_TAU_ mice (Fig. 1(o)). In T2D, the liver exhibited higher levels of gluconeogenesis, consistent with hepatic insulin resistance. Gene expressions of fatty acid synthase (FAS, + 135%; *p* = 0.0147), phosphoenolpyruvate carboxykinase (PEPCK, + 102%; *p* = 0.0001), glucose 6-phosphatase (G6Pase, + 56%; *p* = 0.0085) and fibroblast growth factor 21 (FGF21, + 66%; *p* = 0.0147) were upregulated compared with age-matched PLB_WT_ controls. Overall, these results are indicative of a severe diabetic phenotype in PLB2_TAU_ mice at this age.

### Circadian Activity and Habituation

**Fig. 2:**
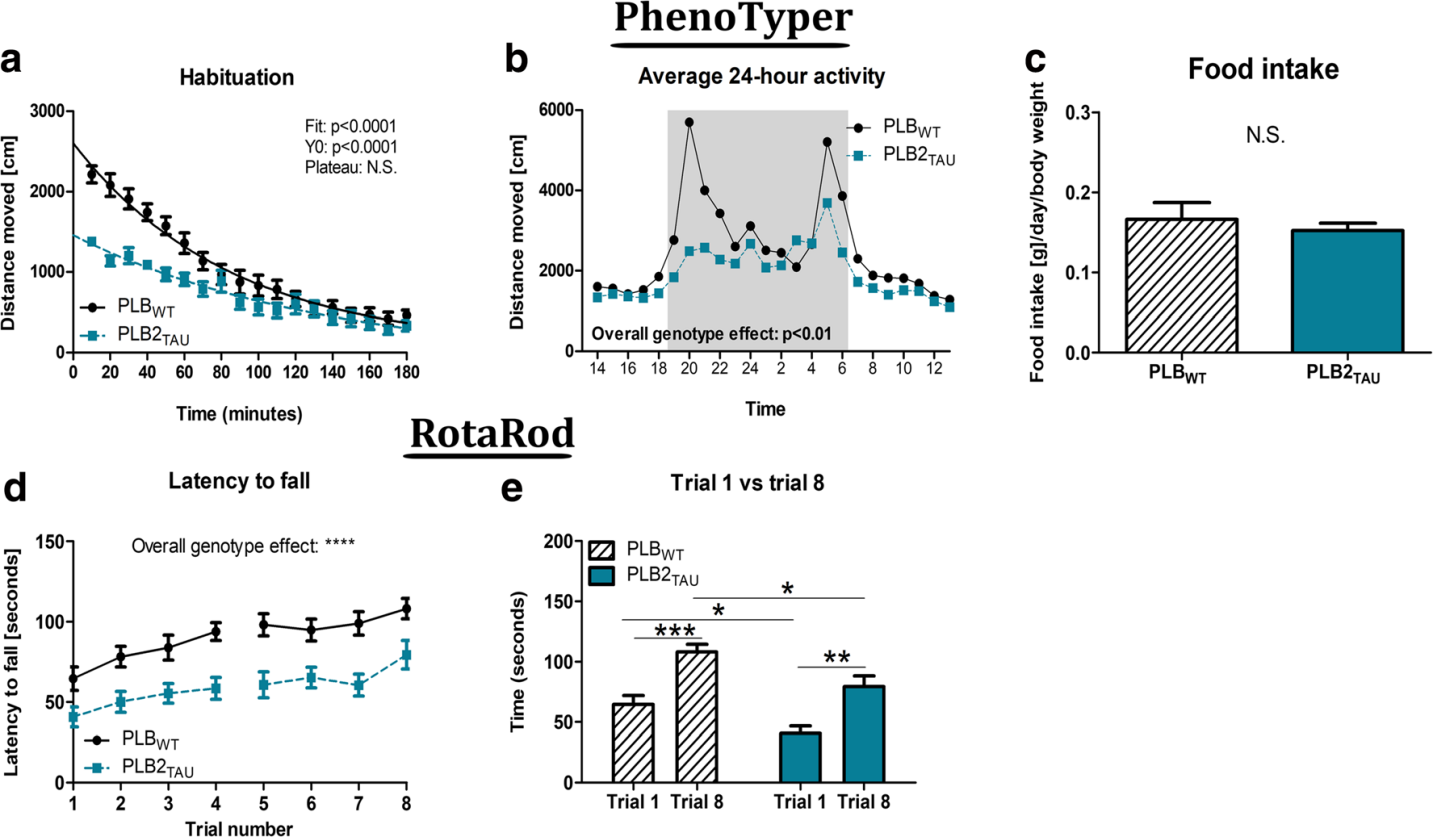
Behavioural data in 8-month-old PLB_WT_ (*n* = 17) and PLB2_TAU_ (*n* = 10) mice during PhenoTyper home cage analysis and RotaRod task. **a** Nonlinear regression analysis (one-phase decay) of activity (distanced moved, cm/10 min) during the 3-h habituation period in the PhenoTyper home cage. **b** Average hourly activity over 24 h. **c** Average food intake per day relative to body weight. **d** Latency to fall from RotaRod was overall reduced in PLB2_TAU_ mice vs PLB_WT_ with both groups displaying motor learning (**e**, trial 1 vs. trial 8). ****p < 0.0001, ***p < 0.001, **p < 0.01, *p < 0.05

Analysis of motor activity over 3 h during habituation to a novel environment revealed a significant difference between genotypes at 8 months of age (Fig. 2(a)): PLB2_TAU_ mice were less active during habituation compared with age-matched controls (*F*(3,642) = 61.25, *p* = 0.0001), particularly during the first hour. Data were fitted to an exponential decay function, which indicated a significant reduction in the initial (Y0) activity of PLB2_TAU_ mice (*p* = 0.0001,) yet no differences in the eventual plateau phase, or rate at which activity declined (*K*). Circadian activity in habituated animals, when considered in mean hourly time bins over 5 consecutive days (Fig. 2(b)), showed the typical circadian and ultradian patterns with higher nocturnal activity profiles in both genotypes. There was however a reduction in the motor activity of PLB2_TAU_ mice compared with the WT; this was especially apparent during the dark phase (8 pm), where only controls showed the typical ultradian nocturnal activity peak. Despite differences in body weight and activity, food intake appeared at comparable levels in transgenic mice vs controls (Fig. 2(c)).

### Motor Performance

Motor coordination and motor learning tests using a RotaRod task identified a significant impairment in motor abilities cf. controls. PLB2_TAU_ mice dismounted faster overall all trials, indicative of a general motor impairment and reduced motor strength (*p* = 0.0001; Fig. 2(c)). Both PLB2_TAU_ (+ 55%; *p* < 0.01) and PLB_WT_ (+ 60%; *p* < 0.001) showed motor learning over the 2-day period with an extended latency to decent over the course of the 8 trials. However, motor performance in trial 8 (*p* < 0.05; Fig. 2(d)) and improvement (trial 1 vs trial 8) was significantly better in WT vs PLB2_TAU_ mice.

### Insulin Signalling in the Brain and Peripheral Tissue

**Fig. 3:**
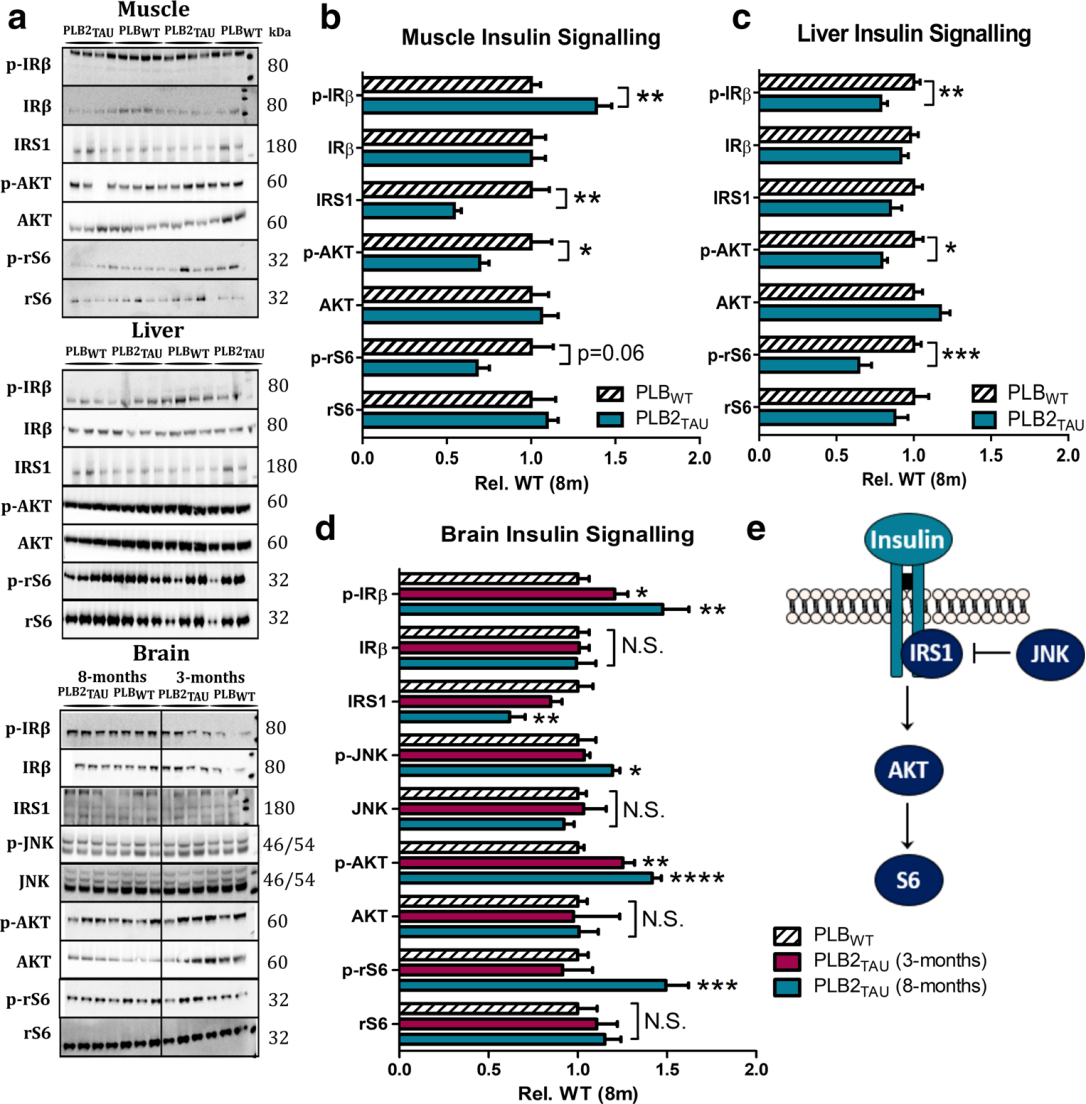
Insulin signalling in the brain vs periphery. (**a**) Representative Western blots for insulin signalling markers in the muscle, liver and brain. Quantification of markers in (**b**) muscle, (**c**) liver and (**d**) brain. PLB_WT_ data for the 3-month group were omitted for clarity, but used for statistical analysis, i.e. each age group was assessed relative to their age-matched PLB_WT_ controls. **e** Simplified schematic showing insulin signalling cascade. All phospho markers are expressed relative to total expression. ****p < 0.0001, ***p < 0.001, **p < 0.01, *p < 0.05

Next, we investigated the molecular basis of the diabetic phenotype of the PLB2_TAU_ mice, by probing for established markers of the insulin signalling pathway (Fig. 3(e)) in brain, muscle and liver tissues, as the major insulin targeting tissues. Insulin resistance, a characteristic feature of T2D, causes diminished downstream effects in target tissues. In liver tissue, PLB2_TAU_ mice presented with a decrease in phosphorylated IRβ (also when expressed relative to total IR) (− 22%, *p* = 0.0031; Fig. 3(c)); there was no change in protein levels of total IRβ, total IRS1, total AKT or total ribosomal S6 (Fig. 3(c)). A decrease in phosphorylated AKT and phosphorylated ribosomal S6 was identified in muscle and liver (− 21%, − 36%; *p* = 0.02 and *p* = 0.0005, respectively).

In contrast to liver, skeletal muscle, a predominant site of insulin-mediated glucose uptake, displayed a significant * increas*e in IRβ phosphorylation relative to total levels (38%, *p* = 0.0010; Fig. 3(b)), with no change in overall total expression. Total IRS1, phosphorylated AKT and phosphorylated ribosomal S6 were decreased, indicating diminished net muscle insulin signalling (− 46%, − 31%, − 33%; *p* = 0.002, *p* = 0.044 and *p* = 0.0640, respectively). Total levels of both AKT and ribosomal S6 remained unchanged (Fig. 3(b)).

IR signalling in the brain does not necessarily follow the changes in peripheral tissue, and indeed we here detected evidence for enhanced brain insulin signalling at 8 months of age, with some changes already occurring at 3 months. For the younger cohort, there was no change in protein levels of IRS1, phosphorylated JNK, total JNK, phosphorylated ribosomal S6 or total ribosomal S6 (Fig. 3(d)) in PLB2_TAU_ brain tissue cf. WTs. However, an increase was detected in phosphorylated IRβ (+ 20%, *p* = 0.041; Fig. 3(d)) and phosphorylated AKT (+ 25%, *p* = 0.002; Fig. 3(d)) at 3 months. At 8 months, a 1.5-fold increase in phosphorylated IRβ emerged in PLB2_TAU_ cf. age-matched WT (*p* = 0.0029; Fig. 3(d)), with total levels remaining unchanged. The downstream substrate IRS1 revealed a decrease in total levels (− 39%, *p* = 0.0067; Fig. 3(d)) but increased levels of both phosphorylated AKT and phosphorylated ribosomal S6 (~ 1.5-fold) were identified for the older age group only (PLB2_TAU_ vs PLB_WT_ controls: *p*’s < 0.001; Fig. 3(d)).

### Phosphorylated Tau and Inflammatory Markers in the Brain

**Fig. 4:**
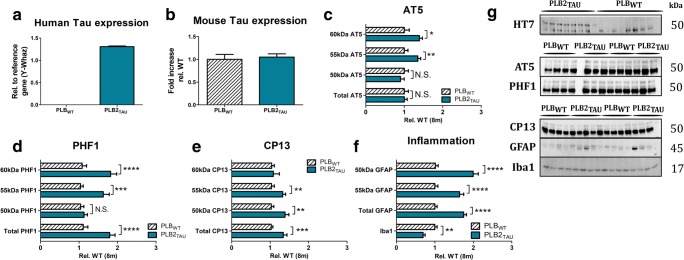
Expression of (phosphorylated) tau and inflammatory markers in 8-month-old PLB_WT_ (*n* = 17) and PLB2_TAU_ (*n* = 10) mice. **a** Quantification of human tau expression via qPCR. **b** Quantification of mouse Tau expression (qPCR). **c** Quantification of individual bands of total Tau (AT5). **d** Quantification of individual bands and total expression for phospho Tau (PHF1) expression relative to total Tau. **e** Quantification of individual bands and total expression for second phospho tau marker (CP13) relative to total Tau. **f** Quantification of 45 kDa, 50 kDa, and total glial fibrillary acid (GFAP, astrocyte marker) and microglia marker Iba1 levels. **g** Representative Western blots of phosphorylated tau and inflammation markers in both PLB_WT_ and PLB2_TAU_ male mice at 8 months of age. ****p < 0.0001, ***p < 0.001, **p < 0.01, *p<0.05

As previously described, PLB2_TAU_ mice express mutated human tau, resulting in increased levels of phosphorylated tau at both PHF-1 and CP-13 epitopes at 6 months of age [17]. We here first compared the expression of mouse and human tau using qPCR. Human tau expression was confirmed in PLB2_TAU_ mice only, with no change in mouse tau expression at 8 months of age between both genotypes (Fig. 4(a, b)). Next, we analysed protein levels of tau phosphorylation in brain tissue of 8-month-old PLB2_TAU_ mice. As in our previous report [17], protein levels of HT7-reactive human tau confirmed an extra band in PLB2_TAU_ mice in the heat-stable fraction, while total tau levels quantified by the AT-5 (3 bands) were unaltered (Fig. 4(c)). When AT5 bands were considered independently, significant increases in protein levels of 55- and 60-kDa tau species were detected (+ 35%, + 39%; *p* = 0.0077, *p* = 0.0143, respectively). Further analysis confirmed elevated levels of PHF-reactive phospho-tau (epitope Ser396/ Ser404) in PLB2_TAU_ mice relative to corresponding PLB_WT_ samples (Fig. 4(c); *p* = 0.0001), with individual bands demonstrating a significant increase (55- and 60-kDa tau species; Fig. 4(c); all *p* < 0.0001). Similar changes in tau phosphorylation were observed for the CP-13 epitope (Ser202) (Fig. 4(d); all *p* < 0.01).

Activation of inflammatory pathways as reported in human FTD was further investigated in brain tissue of PLB2_TAU_ mice by measuring astrocyte (GFAP) and microglia/macrophage (Iba1) markers. Surprisingly, lower levels of ionised calcium-binding adapter molecule 1 (Iba1; Fig. 4(e)) suggested a decrease in activated microglia in PLB2_TAU_ mice compared with WT (*p* = 0.0023; Fig. 4(e)). The astrocyte marker glial fibrillary acid protein (GFAP) (Fig. 4(e)), on the other hand, was overall increased. Specifically, there was a 2-fold increase in the 45-kDa band and 1.6-fold rise of the 50-kDa band (all *p*’s = 0.0001; Fig. 4(e)), indicative of astrogliosis.

### ER Stress in Liver Tissue

**Fig. 5:**
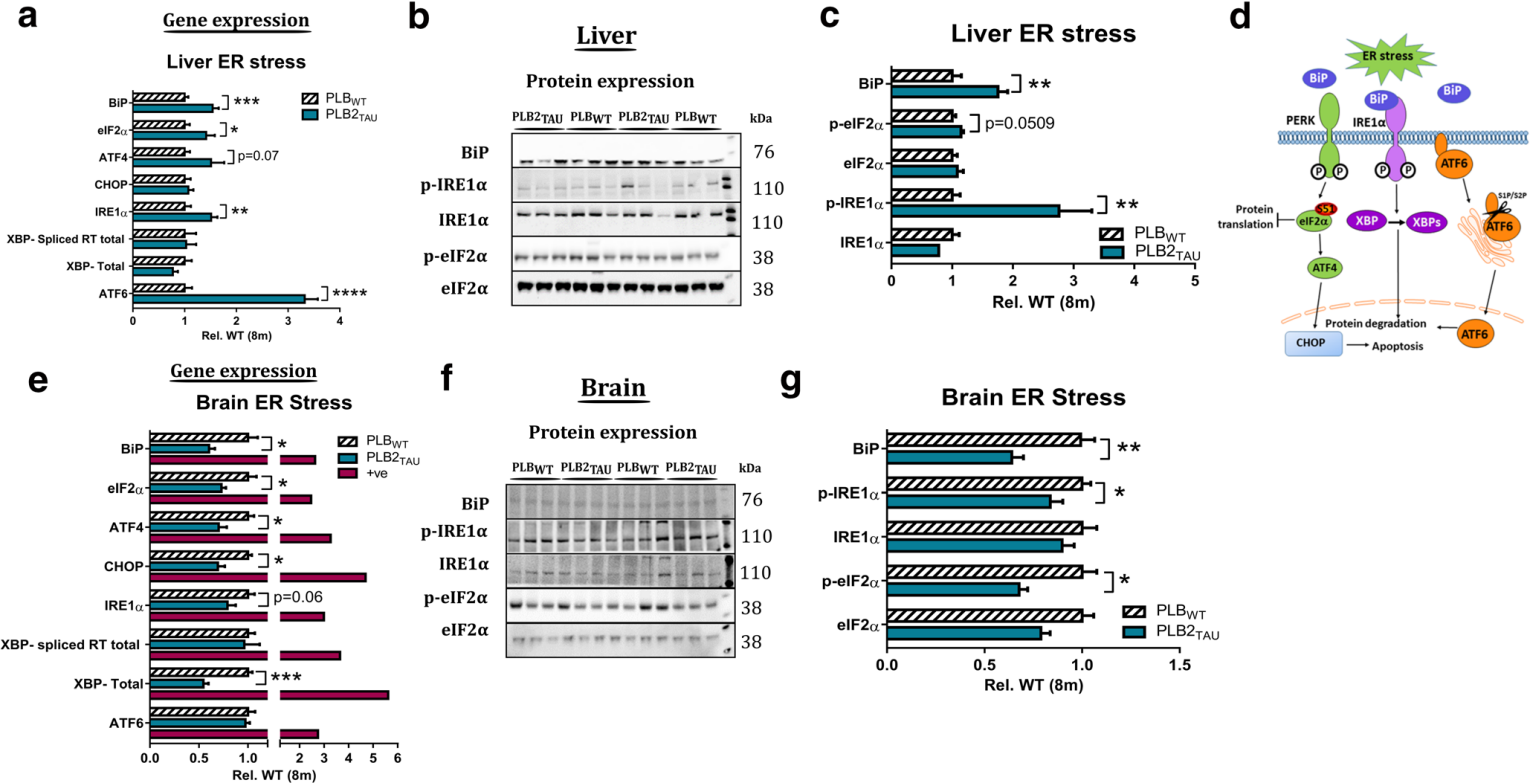
ER stress gene and protein expression in liver and brain tissue of 8-month-old PLB_WT_ (*n* = 17) and PLB2_TAU_ (*n* = 10) mice. **a** Gene expression changes in ER stress–related markers in liver tissue. **b** Western blots of phosphorylated IRE1α, total IRE1α, BiP, phosphorylated eIF2α and total eIF2α in liver tissue. **c** Quantification of ER stress markers in liver tissue. **d** Simplified ER stress signalling cascade. **e** Gene expression changes in brain tissue. Positive control (+ve): thapsigargin-treated primary hippocampal cultures. **f** Western blots of phosphorylated eIF2α, total eIF2α, BiP, phosphorylated IRE1α and total IRE1α in brain tissue. **g** Quantification of ER stress markers in brain tissue. All phospho markers are expressed relative to total expression. *p < 0.05, **p > 0.01, ***p < 0.001 and ****p < 0.0001

As ER stress has also been reported to play a crucial role in peripheral insulin resistance, impaired insulin secretion and the development of T2DM, ER-relevant gene and protein analyses were next investigated in the liver. Here, significantly elevated levels of ER stress markers were detected in PLB2_TAU_ mice (Fig. 5): Gene expressions of *BiP* (+ 54%, *p* = 0.0009); *eIF2α* (+ 41%, *p* = 0.0407), *ATF6* (+ 232%, *p* < 0.0001) and *IRE1α* (+ 51%, *p* = 0.0087) were upregulated compared with age-matched PLB_WT_ controls; there was also a trend for increased levels of *ATF4* (+ 50%, *p* = 0.07). Spliced *XBP1* expression was not changed, and decreased levels of total *XBP1* in PLB2_TAU_ mice compared with controls did not reach significance (− 23%, *p* = 0.08).

Protein analysis in the liver confirmed a significant increase in BiP expression compared with PLB_WT_ controls (+ 75%, *p* = 0.003), alongside phosphorylated IRE1α (+ 175%, *p* = 0.0046). There was also a trend for increased levels of phosphorylated eIF2α (+ 14%, *p* = 0.0509), but no change in total levels of IRE1α or eIF2α (− 23 and − 8%; *p* = 0.091, *p* = 0.5287, respectively).

### ER Stress in Brain Tissue

ER markers were investigated next in brain tissue (Fig. 5). Expression of ER stress–related genes in cortical brain tissue revealed a *decrease* in several markers: The ER chaperone *BiP* (Grp78), bound on the luminal side to the three main transmembrane ER signalling proteins (IRE1α, PERK and ATF6) thus keeping them in an inactive state, was downregulated cf. WT controls (− 49%, *p* = 0.0098). *ATF4* was also significantly decreased in PLB2_TAU_ mice (− 41%, *p* = 0.0054), as was *CHOP*, its downstream regulator of cell death (− 29%, *p* = 0.0065). There was no change in expression of spliced *XBP1* (active form) relative to total levels of *XBP1*; however, total XBP expression was also significantly decreased compared with age-matched controls (− 47%, *p* = 0.0002). For the third UPR arm (ATF6), no change in expression compared with controls was detected (*p* = 0.77).

Protein analysis further confirmed a significant decrease in ER stress markers for BiP expression (− 37%, *p* = 0.004) and phosphorylated activated IRE1α compared with age-matched PLB_WT_ controls (− 18%); total IRE1α protein levels remained unchanged (*p* = 0.04). Phosphorylated eIF2α was also significantly lower (− 34%, *p* = 0.0047). Overall, this suggests a striking down-regulation of two ER stress arms in brain tissue of PLB2_TAU_ mice compared with PLB_WT_ controls.

### Protein Translation Rates

**Fig. 6:**
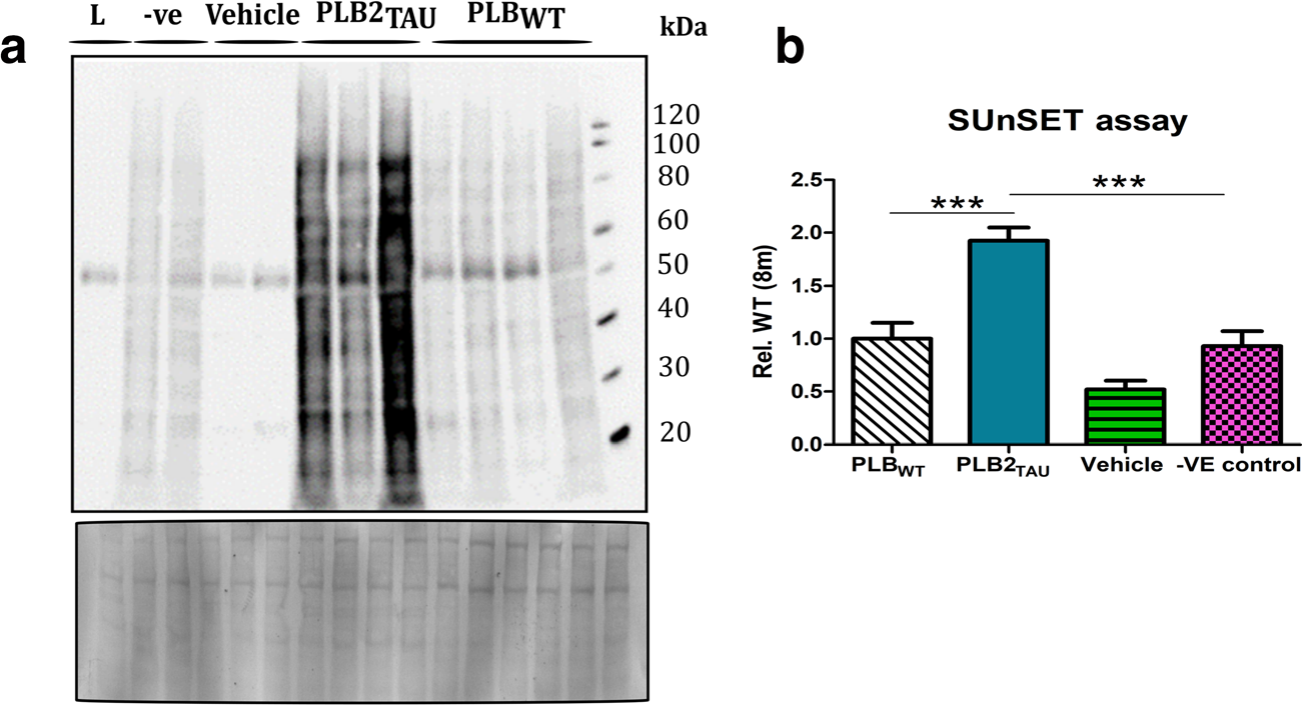
Protein synthesis rates in 8-month-old PLB_WT_ (*n* = 12) and PLB2_TAU_ (*n* = 9) brain tissue. Protein synthesis rates were measured using an adaption to the SUnSET assay. **a** Representative Western blot of puromycin (indicative of newly synthesised proteins), vehicle (10% DMSO in aCSF), negative control (-ve, puromycin and thapsigargin) and the left (L) hemisphere over a 2-h period in both PLB_WT_ and PLB2_TAU_ mice at 8 months of age. **b** Quantification of protein synthesis rates. PLB2_TAU_ mice exhibited an increase in protein translation rates compared to PLB_WT_ controls. ***p < 0.001

The drastic decrease in brain ER stress markers suggested that overall protein translation could be affected (decreased) in PLB2_TAU_ mice. Conversely, it has been previously proposed that an increase in protein turnover can result in a reduction of ER stress–related markers [23]. We therefore next employed a functional readout of ER protein translation in brain tissue. This was achieved via the puromycin assay (SunSeT; [24]), which determined protein synthesis rates in the PLB2_TAU_ mice *in vivo* (Fig. 6)*.* In line with the proposed decreased levels of ER stress markers associated with higher ER demand, and in contrast to the lower activation of UPR pathwyas, the overall protein translation in the PLB2_TAU_ mice was found to be increased at 8 months of age (+ 56%, *p* < 0.001).

## Discussion

Here, we report that PLB2_TAU_ mice with low levels of neuronal human mutated tau expression [17] display the onset of T2D-like symptoms (impaired insulin tolerance) at 3 months of age, gradually progressing with age, where elevated blood insulin levels and impaired glucose handling as well as insulin resistance can be observed at 8 months of age. This phenotype may be due to common pathophysiological mechanisms associated with both T2D and tau pathologies. Although not much is known about the link between tauopathies and insulin resistance in humans, it has been suggested that CNS tau pathology can affect peripheral physiology. One of the key metabolic deficits in T2D is peripheral insulin resistance, where the capacity of insulin to stimulate glucose uptake is impaired. Peripheral metabolic disturbances previously examined in tauopathy mouse models indicated that insulin resistance can promote tau phosphorylation and cognitive decline [25]. In addition, elevated insulin and triglyceride levels have been found in FTD patients [26], and a clinical diagnosis of FTD was associated with a history of T2D [27].

T2D patients also exhibit an increased rate of hepatic gluconeogenesis, resembling the PLB2_TAU_ phenotype [28]. Of note, previous studies have suggested a link between metabolic and motor function [5, 29], though the PLB2_TAU_ phenotype is ultimately more akin to a lean T2D phenotype [30], since it was not associated with increased food intake or body weight. However, the loss of lean body mass (muscle tissue) in the PLB2_TAU_ mice likely contributed to the motor phenotype observed.

Older PLB2_TAU_ mice displayed impaired glucose handling, increased hepatic gluconeogenesis and features of insulin resistance, with the hyperglycaemic state likely a direct result of impaired insulin signalling and responsible for the motor and activity impairments observed, as also proposed by previous studies [5, 29]. Hypoactive traits were also consistent with previous data [17] and most evident during the beginning of the dark (activity) phase. Respective deficits have been reported in other mouse models of FTD [31, 32], although the diabetic status in these models remained unexplored, with phenotypes commonly attributed to tau expression in neuronal motor centres. In patients with spinal and bulbar muscular atrophy, reduced expression of IR and IRS1 has been reported; the resultant motor deficit correlated with the level of insulin dysfunction [33]. A reduction in lean muscle mass has also been reported in early-stage human AD cases [34]; the authors suggested this to be a result of AD pathology or due to a shared pathway between AD and metabolic deficits. Thus, as the metabolic phenotype of PLB2_TAU_ mice is by design a result of brain-specific tau expression, we conclude that peripheral metabolic changes can result as a consequence of tau pathology and are not necessarily the actual primary cause.

A role for tau protein in the regulation of insulin signalling in the brain and periphery has recently been proposed by other studies, largely in line with our data [4, 5, 35], though a complex picture is emerging by closer inspection of recent key publications. Marciniak et al. [5] hypothesised that loss of tau function in tau KO mice impairs brain insulin signalling and results in metabolic dysfunction, demonstrated by a *reduced* hippocampal response to insulin. Here, the opposing changes in insulin signalling in brain vs periphery in PLB2_TAU_ mice are somewhat contrary to the assumption that brain insulin signalling can supress hepatic gluconeogenesis. In agreement with our data, Sajan et al. reported elevated levels of AKT, protein kinase C and phosphorylated tau in brain tissue of insulin-resistant high-fat-fed mice and *ob/ob* mice [36]. They also revealed that *increased* central insulin signalling triggered an *increase* in hepatic gluconeogenesis, glucose intolerance and systemic insulin resistance.

The phenotype of PLB2_TAU_ mice also differs in some aspects to that of THY-Tau22 mice [37], which displayed hyperactivity and hypoinsulinemia but no glucose intolerance, as opposed to hypoactivity and hyperinsulinemia as well as impaired glucose clearance seen in our tau model. Both models have in common an apparent increase in brain IR signalling amidst a lower body weight and altered metabolic status. As THY-Tau22 mice have much higher levels of gene expression and pathology, we suggest that the link between central and peripheral insulin signalling is not necessarily linear and unidirectional, and very likely brain-region and pathology-type specific. Tau’s role in insulin resistance in the low-expression PLB2_TAU_ mice suggests a complex dysregulation rather than a generalised downregulation. Indeed, converging signalling pathways beyond those directly responsible for neuronal IR signalling may be upregulated as a compensatory reaction. Nevertheless, the mutated hTau gene of PLB2_TAU_ mice may be affected due to its hyperphosphorylated state, and this may also affect the principal role of (endogenous) tau as a microtubule-stabilising protein. This is in line with accumulating evidence suggesting a link between tau pathology and metabolic disorders. We therefore here propose that normal tau function is essential to maintain central and peripheral insulin signalling, via a detrimental cycle that implicates neuronal tau in systemic as well as neuronal metabolic regulation.

Despite mounting evidence suggesting a relationship between AD and metabolic disorders, it is not known to what extent the two hallmarks of the disease (amyloid and tau) contribute to peripheral or central metabolic abnormalities. Our data support the possibility that impaired tau function may be crucial for insulin resistance, but it should be noted that metabolic impairments have also been reported in APP-associated mouse models of AD (e.g. APP/PSEN1 mice [38] and 3xTg-AD [39]. Importantly, insulin resistance was more severe in the presence of tau pathology, further confirming a crucial role for tau. Additionally, neuron-specific, low expression of human beta-secretase BACE1 (PLB4 mice) also led to systemic diabetes [18], further confirming that brain-specific, dementia-relevant pathologies can dramatically affect systems physiology.

Peripherally, increased ER stress is a central feature of T2D and insulin resistance [40]. Similar to neurones, pancreatic β-cells are susceptible to ER stress as their ER is highly active due to the secretory demand requiring a high level of quality control. During hyperglycaemia, the sustained demand for insulin results in programmed cell death of β-cells [41]. Of note, *db/db* mice, a common model of insulin resistance, have elevated levels of essential ER markers such as phosphorylated eIF2α and spliced of XBP [41]. This is in accordance with our finding of elevated levels of phosphorylated IRE1α, BiP and increased expression of ATF6 in the liver. Of note, PLB2_TAU_ mice displayed a tissue-specific ER stress profile with for example the ATF6 arm being dramatically affected in liver but not brain. ATF6’s role in liver pathology is in agreement with previously reported links to fatty liver disease and diabetes [42].

Recently, a relationship between neuronal UPR and FTD has been suggested [8, 12], with an upregulation of PERK and eIF2α in both the FTD brain and tauopathy mouse models. Surprisingly, our data showed not only a tissue-specific downregulation of eIF2α but also a reduction in other ER stress–related markers through protein and gene expression analysis in brain tissue (contrasting dramatically with the liver phenotype). In line with our data, reduced brain eIF2α expression had been reported previously in tauopathy mouse model [16]. Here, despite a generalised reduction in ER-related proteins, protein translation was elevated, which agrees with lower eIF2α levels, as phospho-eIF2α inhibits the protein synthesis and controls translation [23]. Elevated protein synthesis may be associated with increased inflammation, as indicated by the raised GFAP levels, in line with neuroinflammation reported in pathologically vulnerable regions in FTD. Heightened astrogliosis in PLB2_TAU_ mice is also in keeping with similar investigations in other tauopathy mouse models [43, 44], as well as the role of astrocytes in brain homeostasis.

The role of microglia in FTD is not as well elucidated [44], and interestingly, our data suggest a potential reduction of Iba1 in PLB2_TAU_ mice. Importantly, this phenotype is in line with reports of reduced levels of microglia in human AD [45] amidst elevated levels of other inflammatory markers such as GFAP and CD88 [46, 47]. The latter is associated with phagocytosis and clearance, while Iba1 is essential for microglia motility and synaptic support [45]; hence, a loss of Iba1 function would be in line with dementia pathology. In addition to this, Streit and colleagues revealed that non-activated microglia colocalised with neuronal structures positive for tau such as neurofibrillary tangles and neuritic plaques [48]. It was suggested that tau pathology does not trigger microglial activation; instead, microglial degeneration and loss of microglial neuroprotection are likely a result of ageing rather than being associated with AD pathology. Recent proposals of opposing M1/M2 phenotypes [49] as well as suggestions of additional (‘dark’) microglia [50] highlight the complexity of microglia (patho-)physiology. These aspects require careful age- and region-specific assessments in tauopathy models in future studies.

Overall, our results suggest a link between neuroinflammation, ER stress, insulin resistance and tau pathology. Subtle neuronal expression of mutated human tau caused inflammation and the dysregulation of ER housekeeping mechanisms, and was sufficient to produce diabetes-like, tissue-specific changes in metabolic regulation. This pathway may offer new avenues for the development of therapeutic interventions.

## Electronic Supplementary Material

ESM 1 (PDF 848 kb)

ESM 2 (DOCX 22 kb)
