# Supplementary material for: Knock-in of Mutated hTAU Causes Insulin Resistance, Inflammation and Proteostasis Disturbance in a Mouse Model of Frontotemporal Dementia
Source: Mol Neurobiol. 2019 Aug 8;57(1):539–50. doi: 10.1007/s12035-019-01722-6 (PMC6968995; doi:10.1007/s12035-019-01722-6)

Muscle: Phospho insulin receptor B and total insulin receptor B

P-IRb

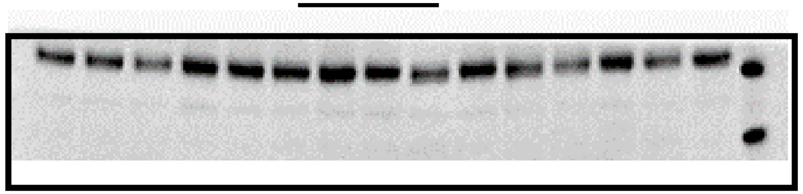

T-IRb

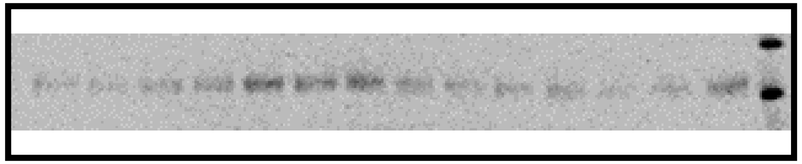

Coomassie

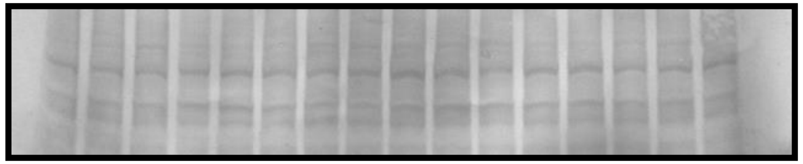

Muscle: Total IRS1

T-IRS1

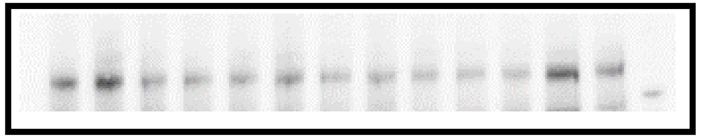

Coomassie

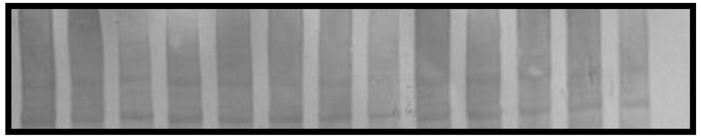

Muscle: Phospho AKT and total AKT

P-AKT

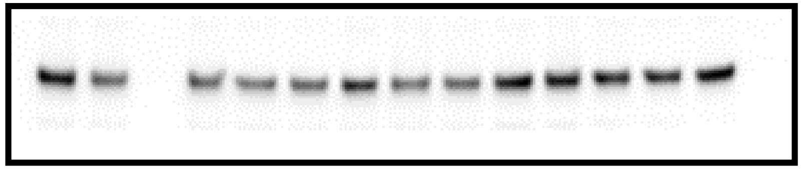

T-AKT

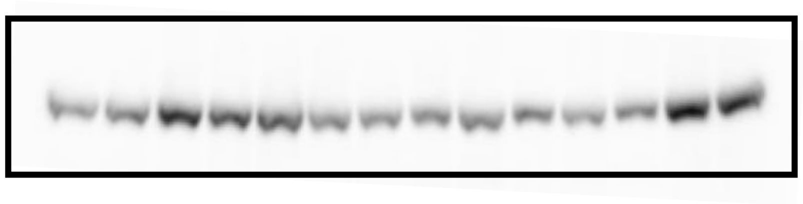

Coomassie

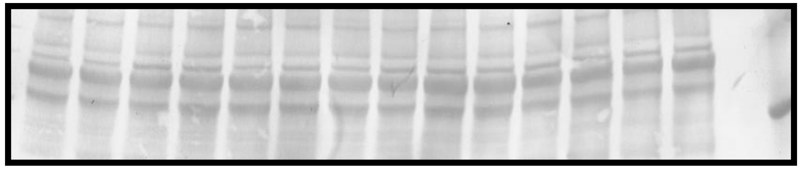

Muscle: Phospho ribosomal S6 and total ribosomal S6

P- ribosomal S6

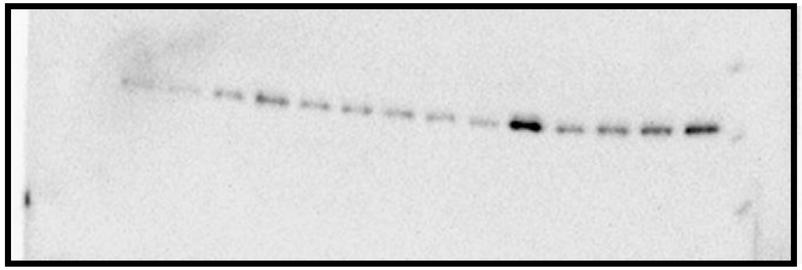

T- ribosomal S6

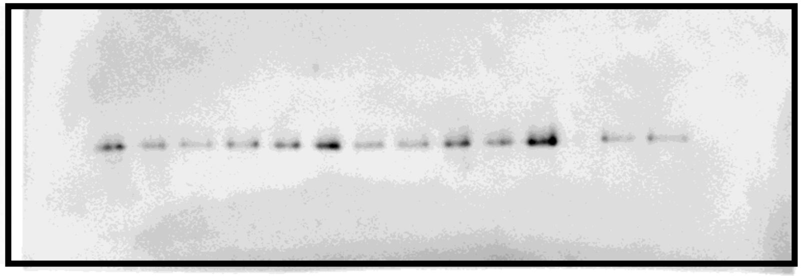

Coomassie

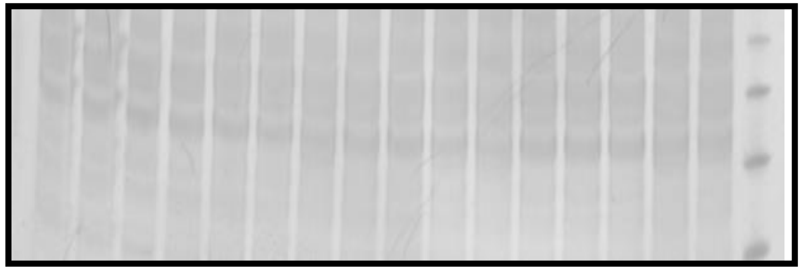

Liver: Phospho insulin receptor B and total insulin receptor B

P-IRb

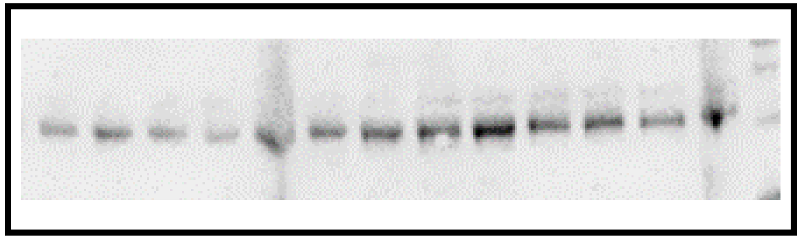

T-IRb

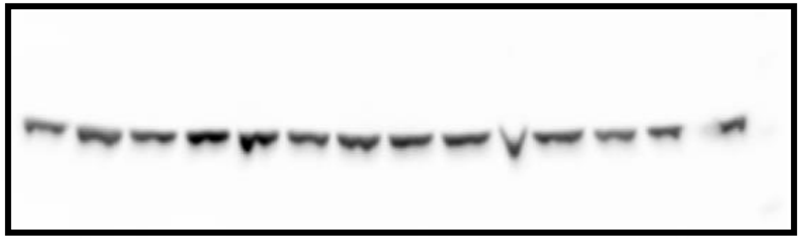

Coomassie

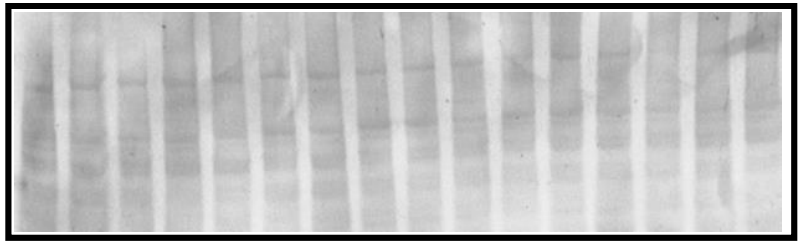

Liver: Total IRS1

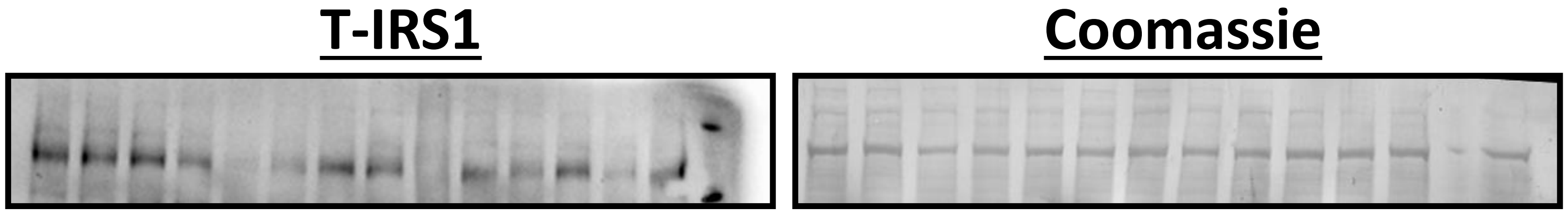

Liver: Phospho AKT and total AKT

p-AKT

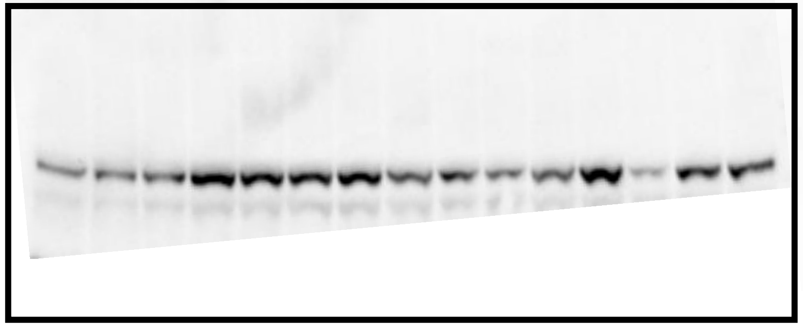

T-AKT

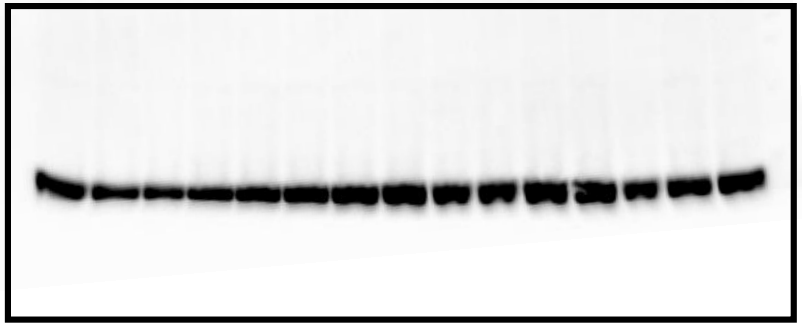

Coomassie

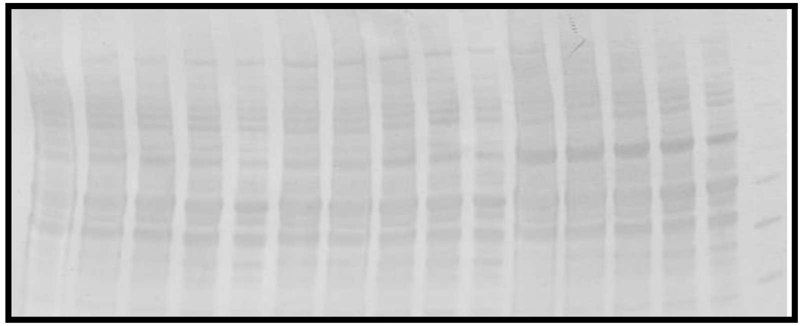

Liver: Phospho ribosomal S6 and total ribosomal S6

P- ribosomal S6

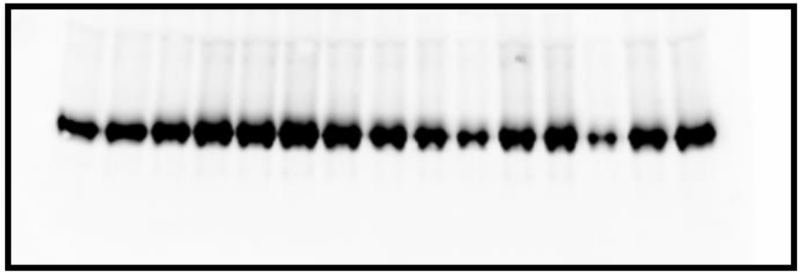

T- ribosomal S6

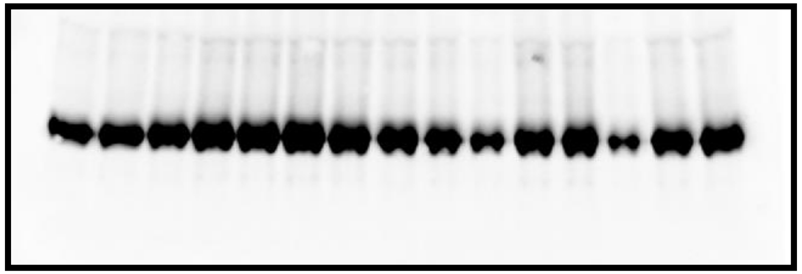

Coomassie

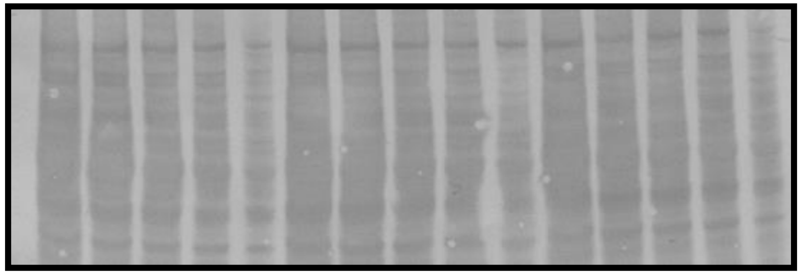

Brain: Phospho insulin receptor B and total insulin receptor B - 3 vs 8-months

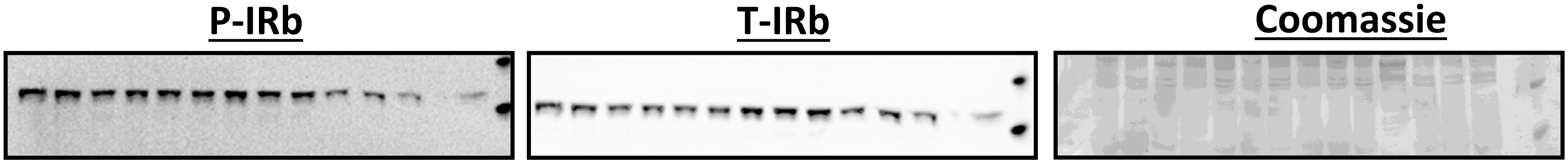

Brain: Total IRS1 3 vs 8-months

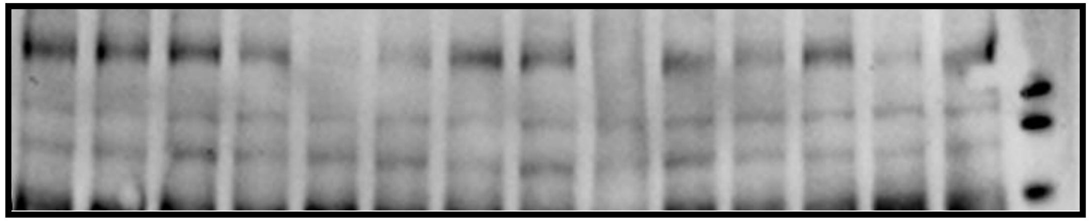

Coomassie

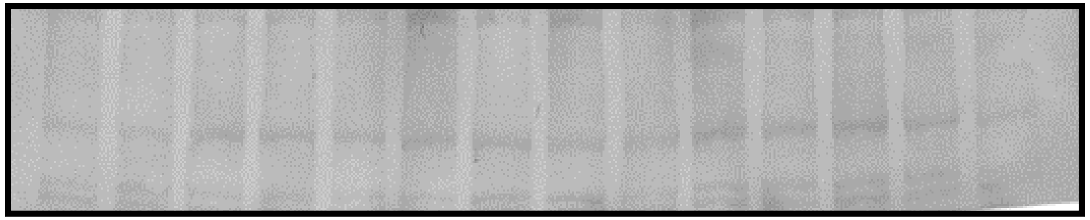

Brain: Phospho JNK and total JNK - 3 vs 8-months

p-JNK

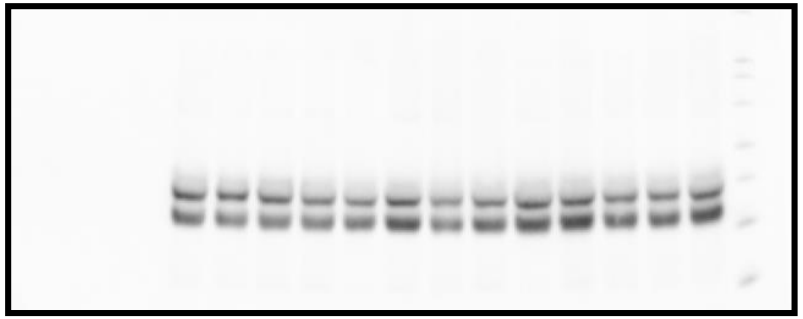

T-JNK

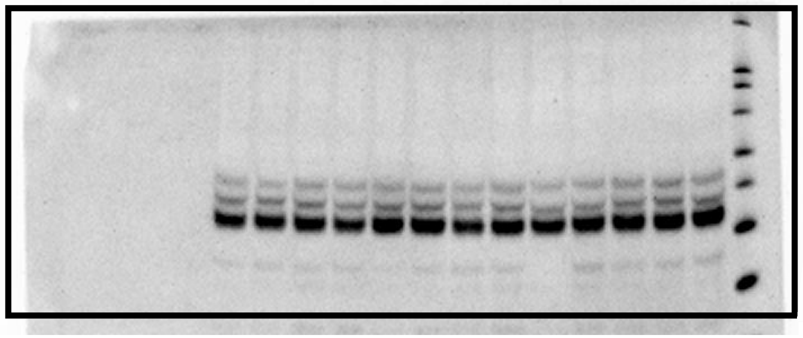

Coomassie

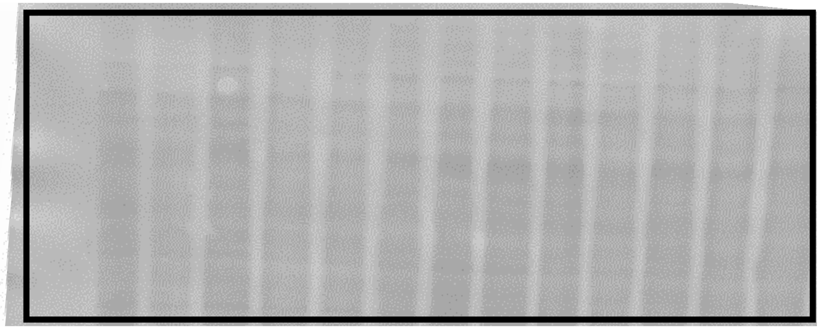

Brain: Phospho AKT and total AKT 3 vs 8-months

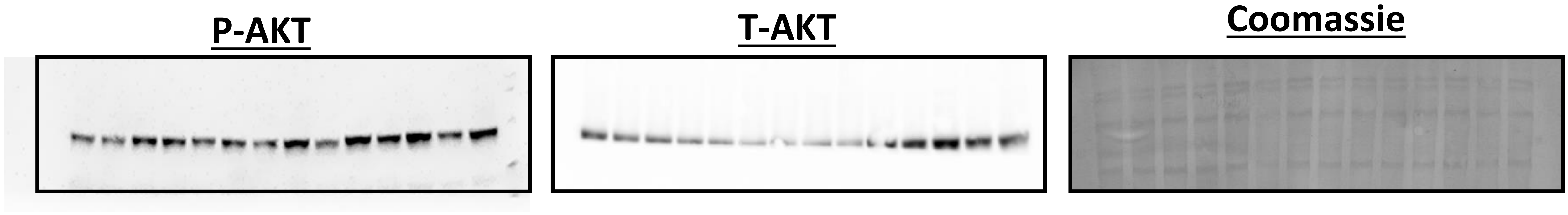

Brain: Phospho ribosomal S6 and total ribosomal S6 -3 vs 8-months

p-ribosomal S6

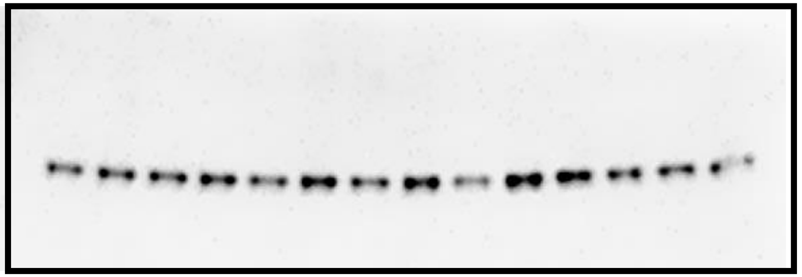

T-Ribosomal S6

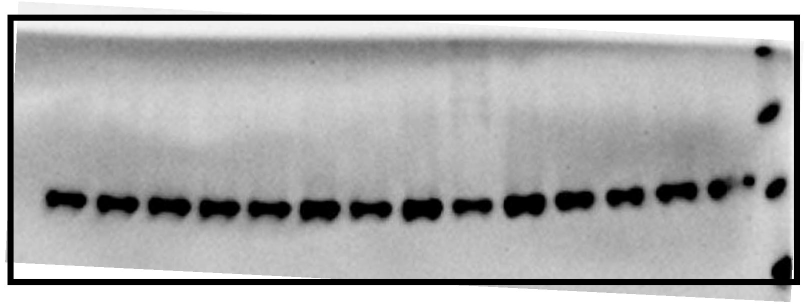

Coomassie

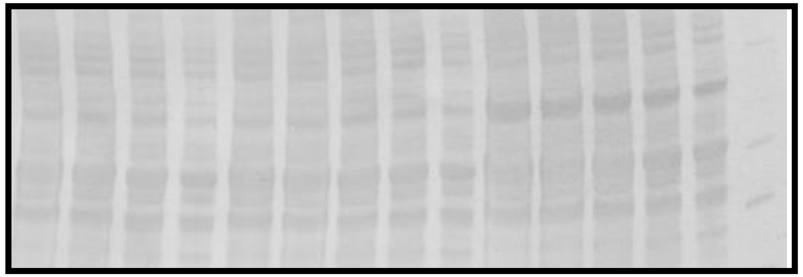

Brain: HT7

HT7

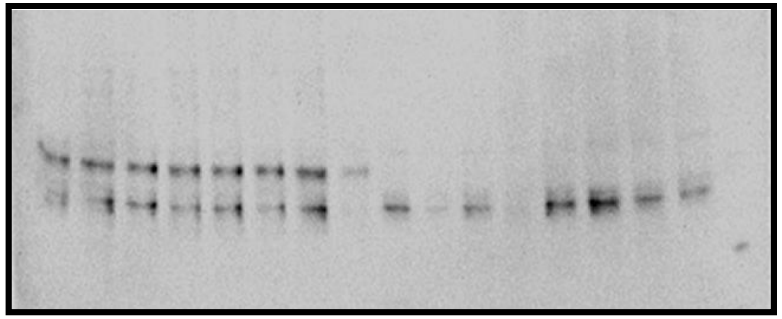

AT5

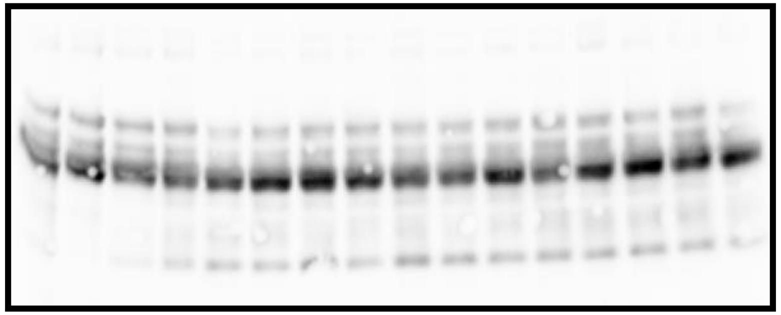

Brain: PHF 1 & AT5

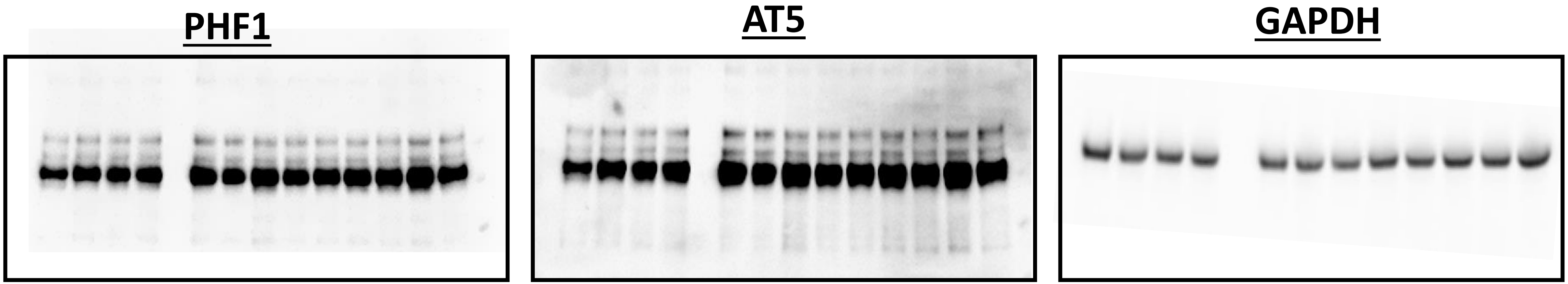

Brain: CP13 & AT5

CP13

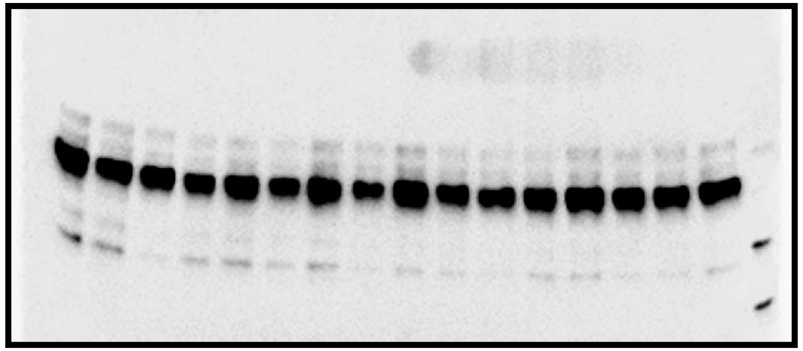

AT5

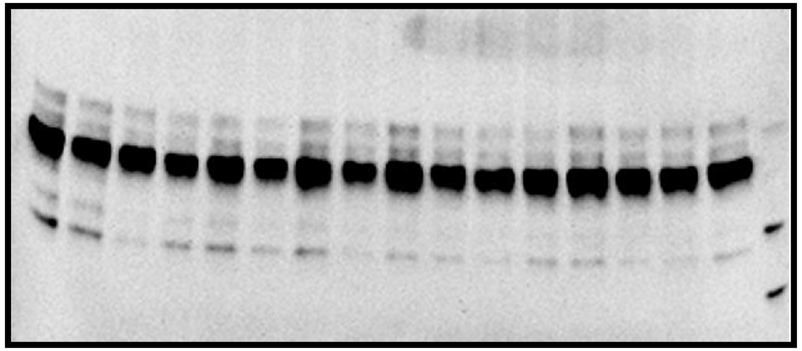

GAPDH

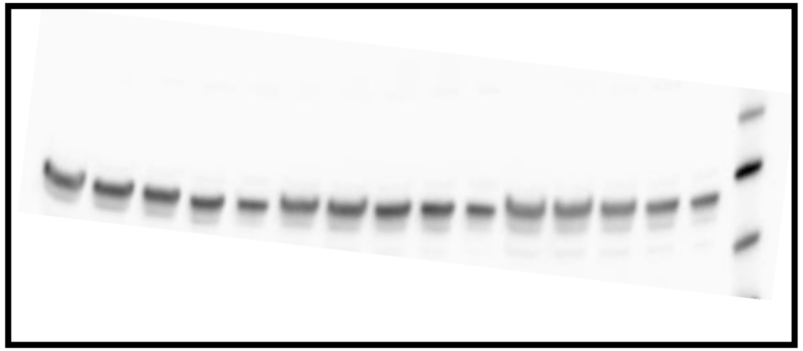

Brain: GFAP

GFAP

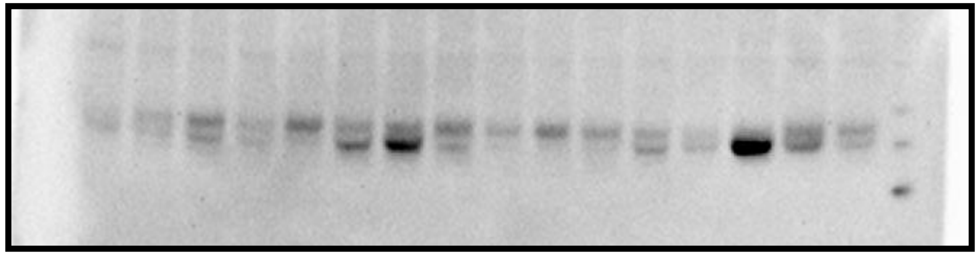

Coomassie

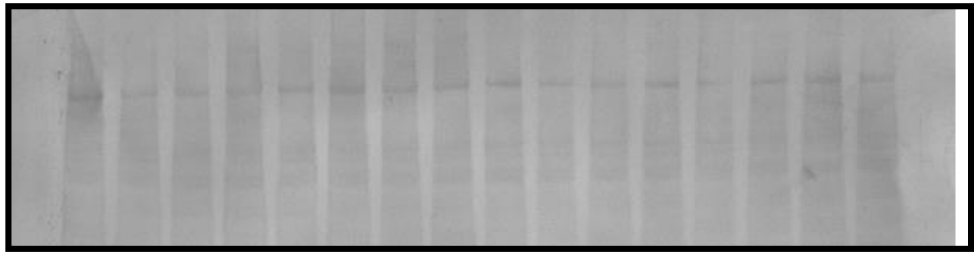

Brain: IBA1

IBA1

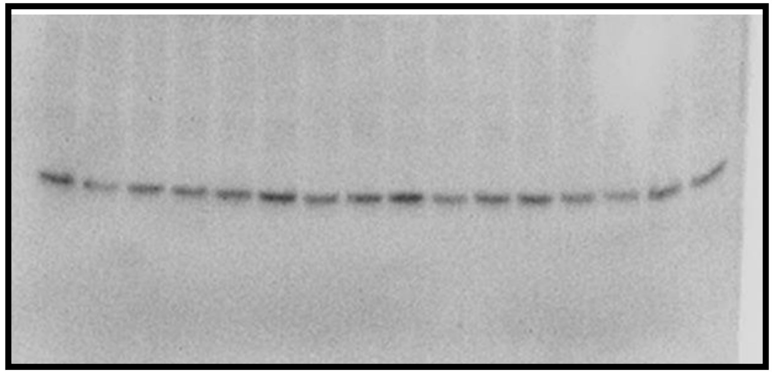

Coomassie

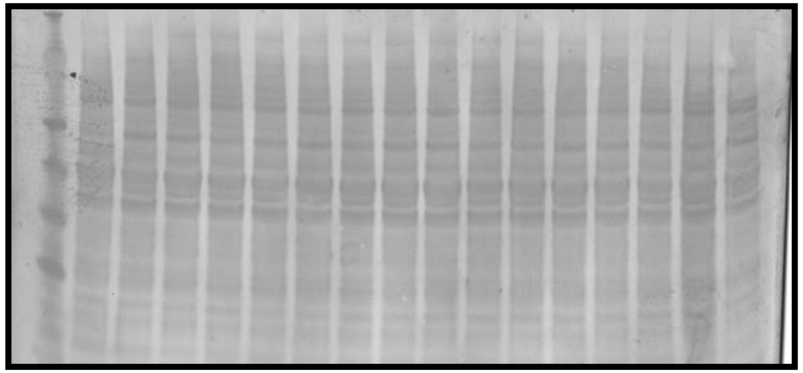

Liver: BIP

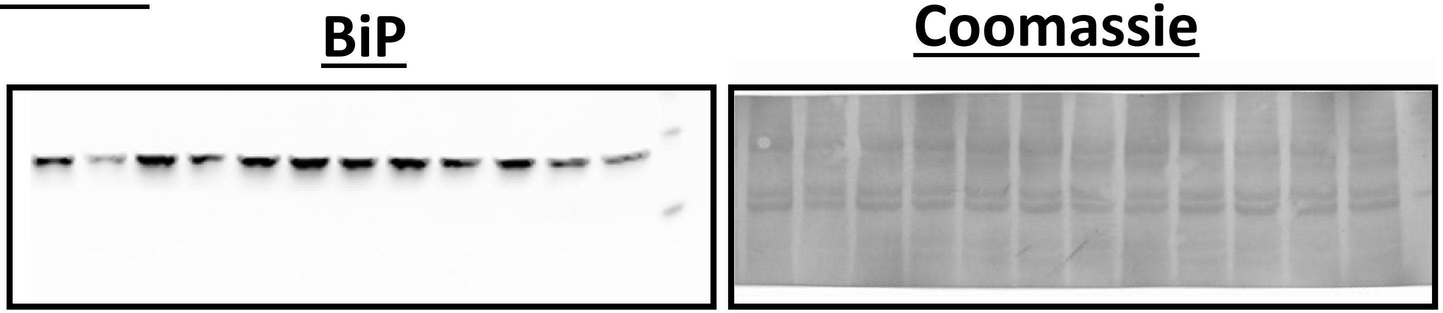

Liver: Phospho- IRE & total IRE

p-IRE1a

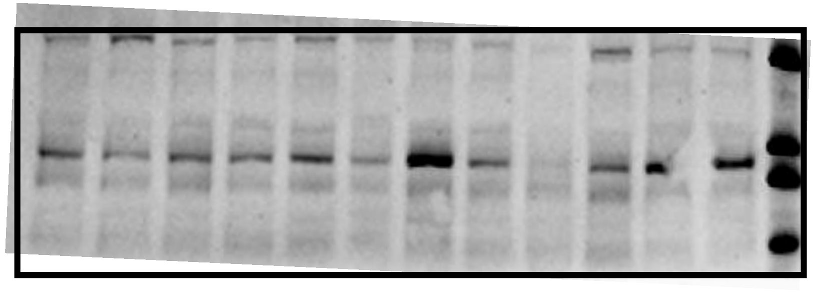

T-IRE1a

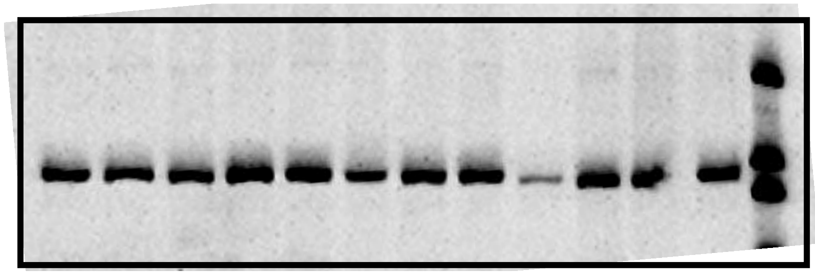

Coomassie

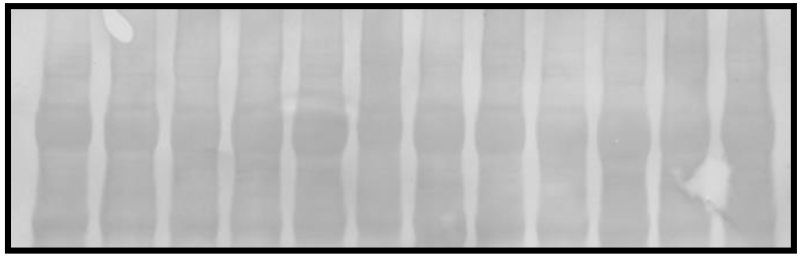

Liver: Phospho- eif2a & total eIF2a

P-eIF2a

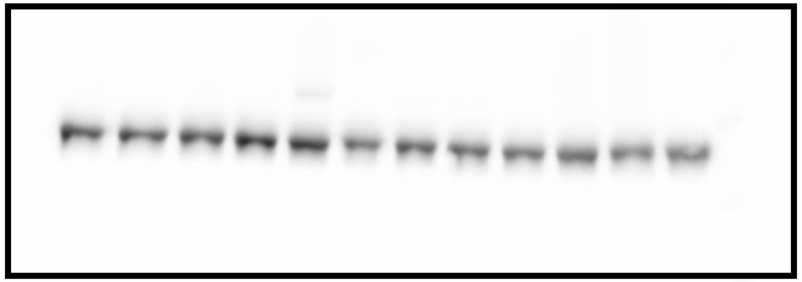

T-eIF2a

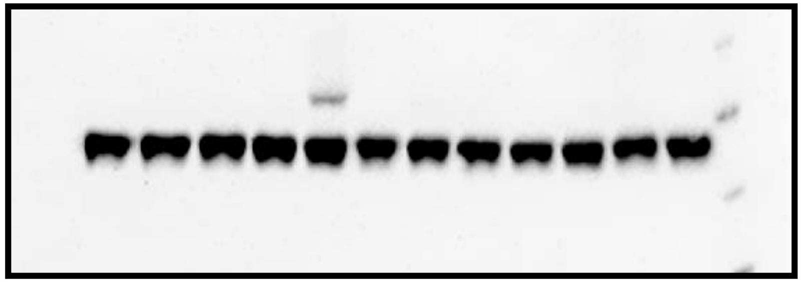

Coomassie

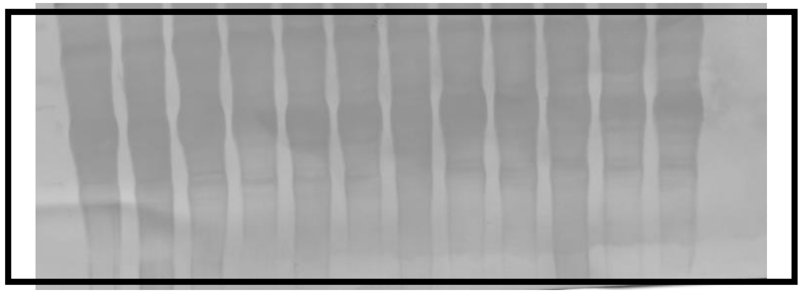

Brain: BIP

BiP

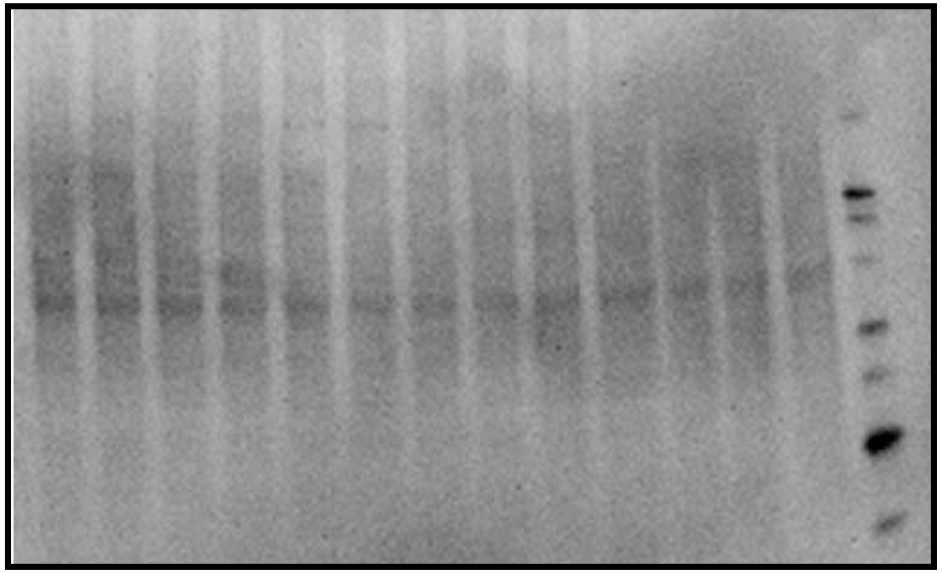

Coomassie

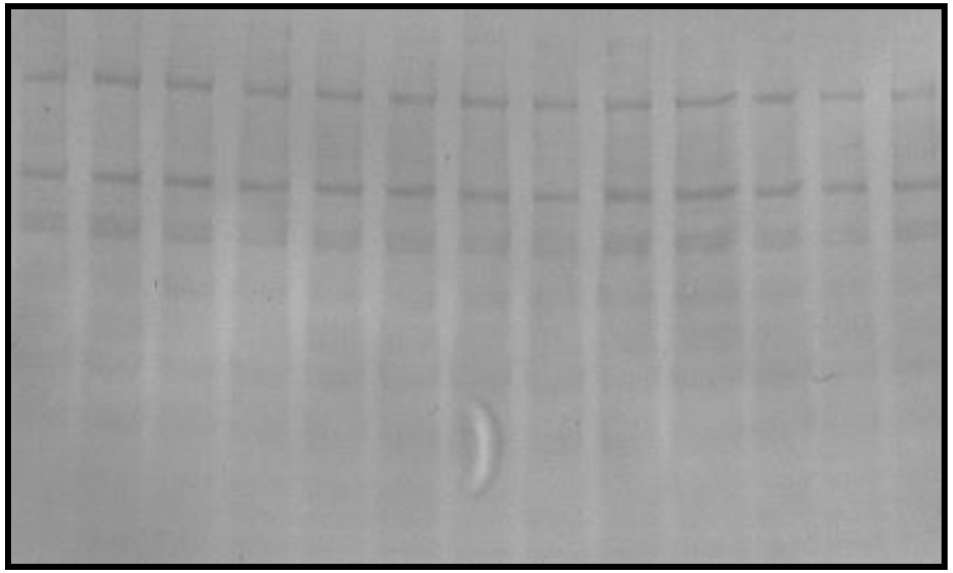

Brain: Phospho- IRE & total IRE

p-IRE1a

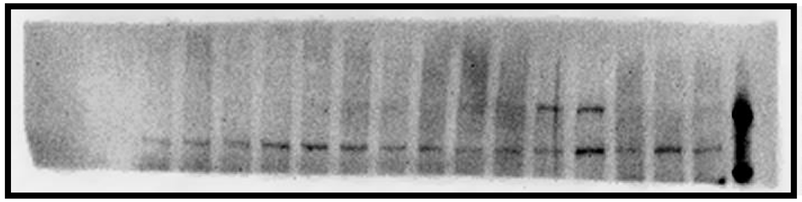

T-IRE1a

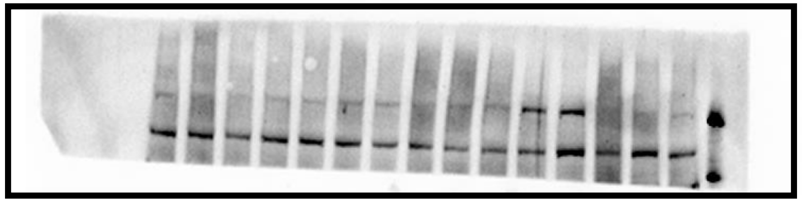

Coomassie

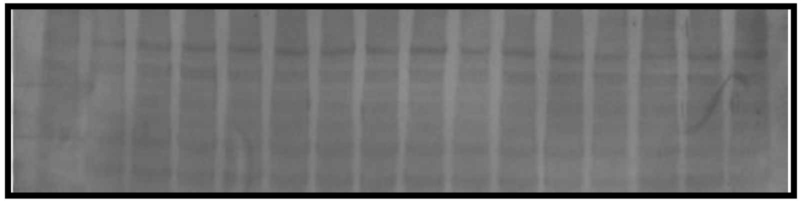

Brain: Phospho- eif2a & total eIF2a

P-eIF2a

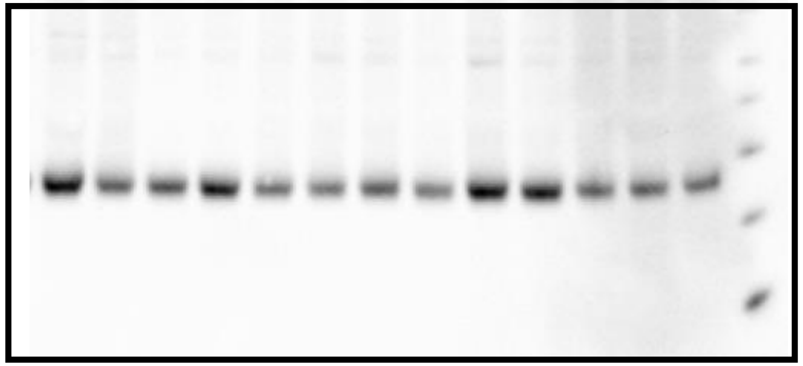

T-eIF2a

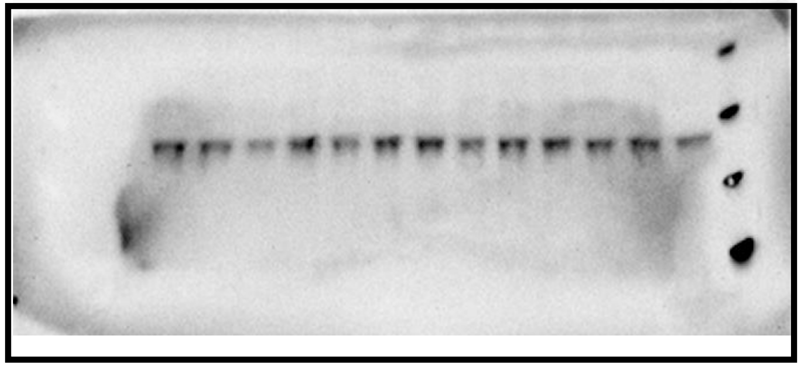

Coomassie

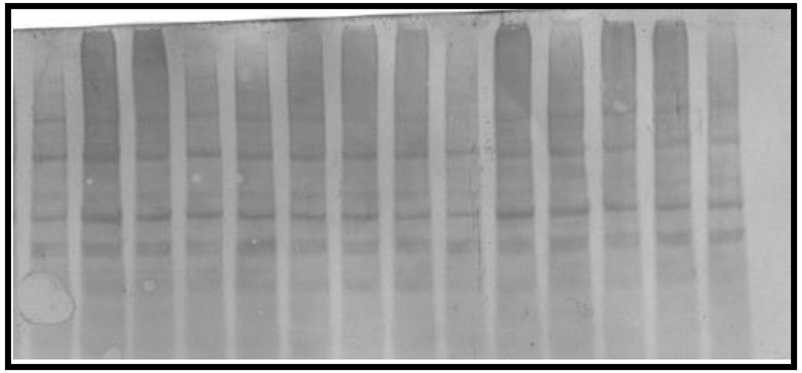

Puromycin

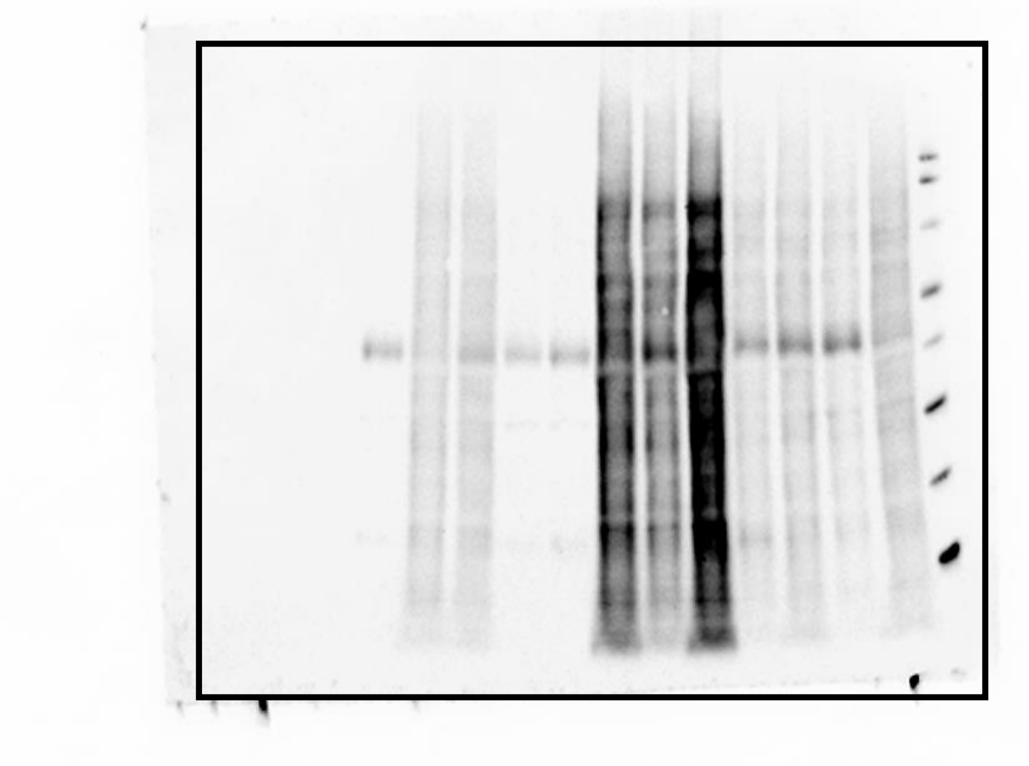

Coomassie

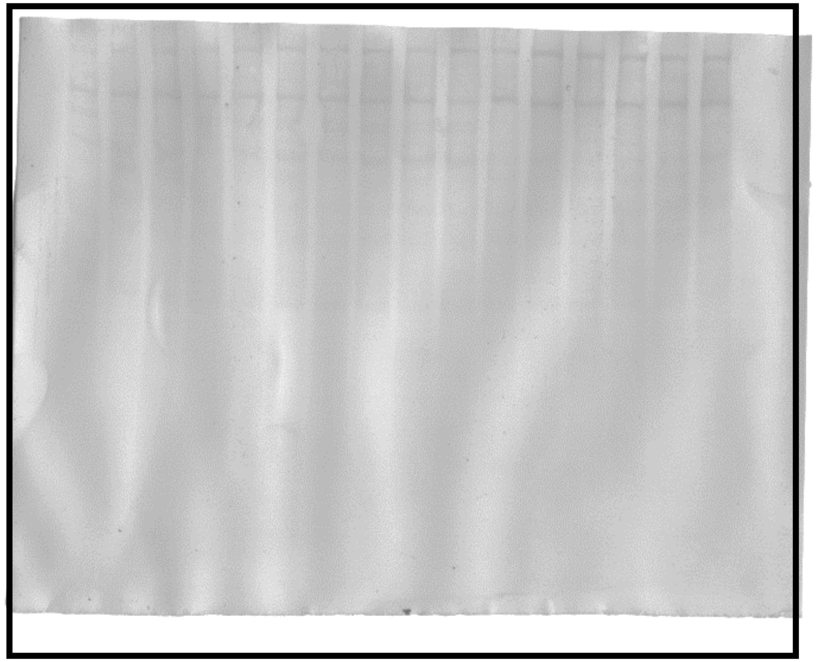

Supplement: Supplementary file 1 — (PDF 848 kb) [file 12035_2019_1722_MOESM1_ESM.pdf]
