# Supplementary material for: Knock-in of Mutated hTAU Causes Insulin Resistance, Inflammation and Proteostasis Disturbance in a Mouse Model of Frontotemporal Dementia
Source: Mol Neurobiol. 2019 Aug 8;57(1):539–50. doi: 10.1007/s12035-019-01722-6 (PMC6968995; doi:10.1007/s12035-019-01722-6)
Supplement: Supplementary file 2 — (DOCX 22 kb) [file 12035_2019_1722_MOESM2_ESM.docx]

# Supplementary material

| **Antibody** | **Supplier** | **Concentration** | **Secondary** | **Secondary concentration** | **Supplier** |
| --- | --- | --- | --- | --- | --- |
| **p-eIF2α** | Cell Signalling | 1:500 | Goat anti-rabbit | 1:5000 | Merck Millipore |
| **Total-eIF2α** | Cell Signalling | 1:500 | Goat anti-rabbit | 1:5000 | Merck Millipore |
| **BiP** | Cell Signalling | 1:1000 | Goat anti-rabbit | 1:5000 | Merck Millipore |
| **p-IRE1α** | Thermo-Fisher | 1:1000 | Goat anti-rabbit | 1:5000 | Merck Millipore |
| **Total IRE1α** | Cell Signalling | 1:1000 | Goat anti-rabbit | 1:5000 | Merck Millipore |
| **HT-7** | Pierce | 1:1000 | Rabbit anti-mouse | 1:5000 | Merck Millipore |
| **AT-5** | Abcam | 1:1000 | Rabbit anti-mouse | 1:5000 | Merck Millipore |
| **PHF-1** | Gift from Professor Peter Davies | 1:500 | Rabbit anti-mouse | 1:5000 | Merck Millipore |
| **CP-13** | Gift from Professor Peter Davies | 1:500 | Rabbit anti-mouse | 1:5000 | Merck Millipore |
| **GFAP** | Sigma-Aldrich | 1:1000 | Rabbit anti-mouse | 1:5000 | Merck Millipore |
| **IBA1** | Wako Pure Chemical industries | 1:200 | Goat anti-rabbit | 1:5000 | Merck Millipore |
| **p-IRβ** | Santa Cruz | 1:1000 | Goat anti-rabbit | 1:5000 | Merck Millipore |
| **Total IRβ** | Cell Signalling | 1:1000 | Goat anti-rabbit | 1:5000 | Merck Millipore |
| **Total IRS1** | Cell Signalling | 1:1000 | Goat anti-rabbit | 1:5000 | Merck Millipore |
| **p-AKT** | Cell Signalling | 1:1000 | Goat anti-rabbit | 1:5000 | Merck Millipore |
| **Total AKT** | Cell Signalling | 1:1000 | Goat anti-rabbit | 1:5000 | Merck Millipore |
| **p-rS6** | Cell Signalling | 1:1000 | Goat anti-rabbit | 1:5000 | Merck Millipore |
| **Total rS6** | Cell Signalling | 1:1000 | Goat anti-rabbit | 1:5000 | Merck Millipore |
| **p-JNK** | Cell Signalling | 1:1000 | Goat anti-rabbit | 1:5000 | Merck Millipore |
| **Total JNK** | Cell Signalling | 1:1000 | Goat anti-rabbit | 1:5000 | Merck Millipore |

**Supplementary Table 1:** Antibodies used for protein expression quantification, “p-“= phospho-specific

| **Gene** | **Primer sequence (5’ to 3’)** | | **Tm** | **Supplier** |
| --- | --- | --- | --- | --- |
| BetaActin | **F** | GATCTGGCACCACACACCTTC | 66.9 | Sigma Aldrich |
|  | **R** | GGGGTGTTGAAGGTCTCAAA | 63.9 |  |
| GAPDH | **F** | TGACCACAGTCCATGCCATC | 67.3 |  |
|  | **R** | GACGGACACATTGGGGGTAG | 66.4 |  |
| Y-Whaz | **F** | GAAAAGTTCTTGATCCCCAATGC | 66.5 |  |
|  | **R** | TGTGACTGGTCCACAATTCCTT | 65.5 |  |
| 18S | **F** | CTCTGTTCCGCCTAGTCCTG | 63.9 |  |
|  | **R** | AATGAGCCATTCGCAGTTTC | 64.0 |  |
| NoNo | **F** | GCCAGAATGAAGGCTTGACTAT | 63.3 |  |
|  | **R** | TATCAGGGGGAAGATTGCCCA | 69.4 |  |
| ATF4 | **F** | ATGACCGAAATGAGCTTCCT | 56.9 |  |
|  | **R** | CTGGAGAACCCATGAGGTTTG | 58.5 |  |
| Chop | **F** | CCACGGGACCTACTACGAGT | 60.4 |  |
|  | **R** | ACGGCAAAGAGATCGGAGAGA | 60.9 |  |
| BiP | **F** | TTCAGCCAATTATCAGCAAACTCT | 58.9 |  |
|  | **R** | TTTTCTGATGTATCCTCTTCACCAGT | 60.0 |  |
| XBP-Spliced | **F** | AAACAGAGTAGCAGCGCAGACTGC | 65.6 |  |
|  | **R** | TCCTTCTGGGTAGACCTCTGGGAG | 64.3 |  |
| XBP- total | **F** | AAGAACACGCTTGGGAATGG | 58.8 |  |
|  | **R** | ACTCCCCTTGGCCTCCAC | 60.9 |  |
| ATF6 | **F** | TTATCAGCATACAGCCTGCG | 63.8 |  |
|  | **R** | CTTGGGACTTTGAGCCTCTG | 63.9 |  |
| Human TAU | **F** | CAGGAGTTCGAAGTGATGGAAGA | 66.5 |  |
|  | **R** | AGCCCCCCTGATCTTTCCT | 66.0 |  |
| Mouse TAU | **F** | ACCATAGAGTTTGGGATGGA | 60.8 |  |
|  | **R** | GCTTCCTCCTAGTCTCCACA | 61.0 |  |
| PEPCK | **F** | GAGATAGCGGCACAA | 53.3 |  |
|  | **R** | TTCAGAGACTATGCGGTG | 57.3 |  |
| G6Pase | **F** | CCTGGTTTTAGGAGCCGTGT | 65.1 |  |
|  | **R** | ATGGTGCTGAAGGGTAAGCC | 65.2 |  |
| FGF21 | **F** | ACCTGGAGATCAGGGAGGAT | 63.8 |  |
|  | **R** | CACCCAGGATTTGAATGACC | 64.1 |  |
| F.A.S | **F** | TGCTCCCAGCTGCAGGC | 69.5 |  |
|  | **R** | GCCCGGTAGCTCTGGGTGTA | 68.4 |  |

**Supplementary Table 2:** List of primers used for qPCR
